# BPAG1a and b Associate with EB1 and EB3 and Modulate Vesicular Transport, Golgi Apparatus Structure, and Cell Migration in C2.7 Myoblasts

**DOI:** 10.1371/journal.pone.0107535

**Published:** 2014-09-22

**Authors:** Kseniia Poliakova, Adijat Adebola, Conrad L. Leung, Bertrand Favre, Ronald K. H. Liem, Isabelle Schepens, Luca Borradori

**Affiliations:** 1 Department of Clinical Research, University of Bern, Bern, Switzerland; 2 Graduate School for Cellular and Biomedical Sciences, University of Bern, Bern, Switzerland; 3 Department of Dermatology, Inselspital, Bern University Hospital, Bern, Switzerland; 4 Department of Pathology and Cell Biology, Columbia University College of Physicians and Surgeons, New York, New York, United States of America; Stanford University School of Medicine, United States of America

## Abstract

BPAG1a and BPAG1b (BPAG1a/b) constitute two major isoforms encoded by the dystonin (*Dst*) gene and show homology with MACF1a and MACF1b. These proteins are members of the plakin family, giant multi-modular proteins able to connect the intermediate filament, microtubule and microfilament cytoskeletal networks with each other and to distinct cell membrane sites. They also serve as scaffolds for signaling proteins that modulate cytoskeletal dynamics. To gain better insights into the functions of BPAG1a/b, we further characterized their C-terminal region important for their interaction with microtubules and assessed the role of these isoforms in the cytoskeletal organization of C2.7 myoblast cells. Our results show that alternative splicing does not only occur at the 5′ end of *Dst* and *Macf1* pre-mRNAs, as previously reported, but also at their 3′ end, resulting in expression of additional four mRNA variants of BPAG1 and MACF1. These isoform-specific C-tails were able to bundle microtubules and bound to both EB1 and EB3, two microtubule plus end proteins. In the C2.7 cell line, knockdown of BPAG1a/b had no major effect on the organization of the microtubule and microfilament networks, but negatively affected endocytosis and maintenance of the Golgi apparatus structure, which became dispersed. Finally, knockdown of BPAG1a/b caused a specific decrease in the directness of cell migration, but did not impair initial cell adhesion. These data provide novel insights into the complexity of alternative splicing of *Dst* pre-mRNAs and into the role of BPAG1a/b in vesicular transport, Golgi apparatus structure as well as in migration in C2.7 myoblasts.

## Introduction

Bullous pemphigoid antigen 1 (BPAG1), encoded by the dystonin gene (*Dst*), is a member of the plakin family of cytolinkers. This protein family connects the intermediate filament (IF), microtubule (MT) and microfilament (MF) cytoskeletal networks with each other and to distinct cell membrane sites and act as scaffolds and adaptors for signaling proteins that modulate cytoskeletal dynamics, cell migration, differentiation, and stress responses [1]–[3].

Multiple transcription initiation sites and alternative splicing of *Dst* results in three major BPAG1 isoforms, BPAG1a (∼600 kDa), BPAG1b (∼800 kDa), and BPAG1e (∼300 kDa), which exhibit different tissue-specific expression profiles and functions. Furthermore, at least three alternative transcription start sites give rise to several *Dst* mRNA variants encoding different N-terminal BPAG1a/b isoforms [4]. While BPAG1e is found in stratified epithelia, BPAG1a and b are predominantly expressed in neurons and in striated muscles, respectively [4], [5]. BPAG1a/b are homologous to the mammalian microtubule actin cross-linking factor 1 (MACF1) isoforms a and b (MACF1a/b) [6], and to *Drosophila* Short stop (Shot) [7]. MACF1a and Shot are important for MT network structure maintenance [8], [9]. Shot, BPAG1a/b and MACF1a/b differ from the other plakins by having a unique rod domain that consists of spectrin repeats (SRs), in addition to the SRs that make up the common plakin domain. These proteins are therefore also called spectraplakins [1].

**Figure 1:**
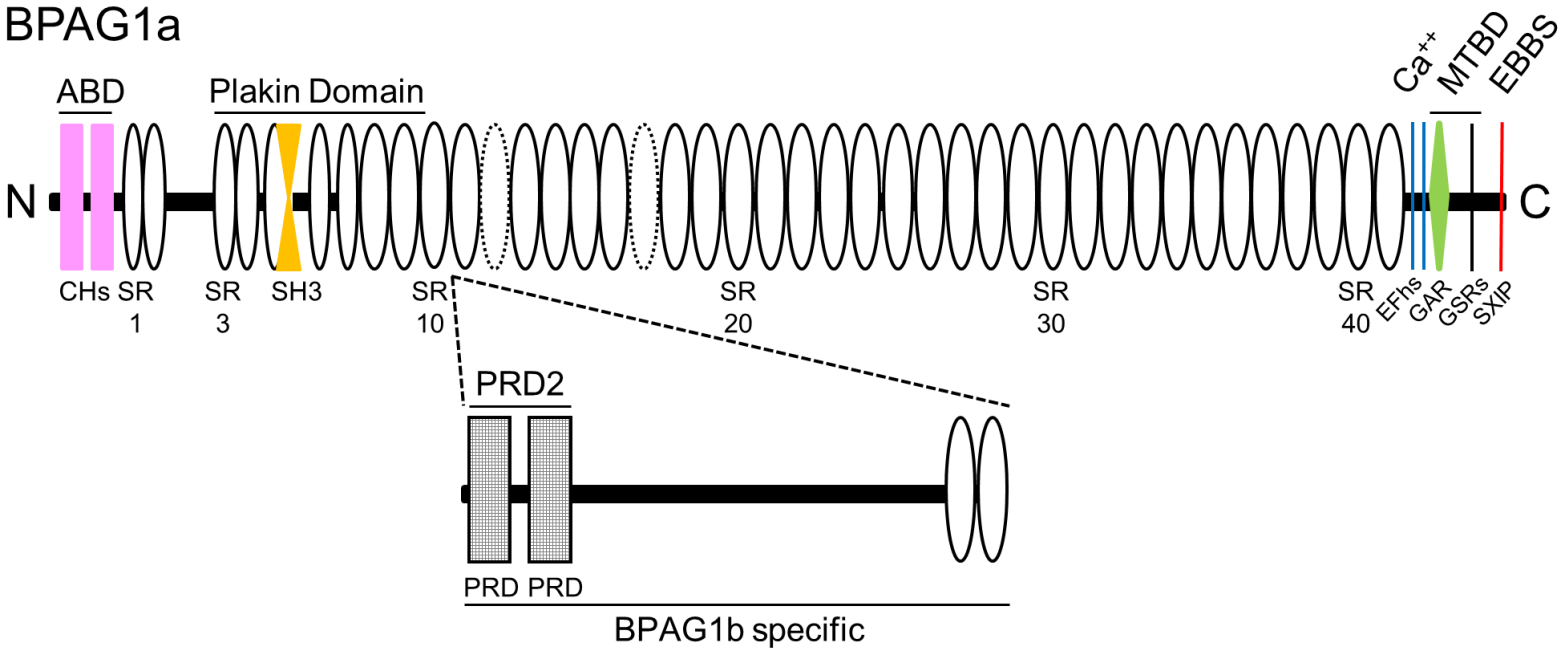
Schematic representation of BPAG1a and b domain organization. ABD, actin-binding domain; CH, calponin homology domain; SR, spectrin repeat (dotted ovals represent putative SRs, not previously identified as SRs [63], or predicted in mouse or human BPAG1a sequence by SMART [64]; some SRs in the plakin domain were deduced from the alignment with plectin plakin domain [65], SH3, src homology-3 domain (is atypical and embedded in SR5 [66]); PRD, plakin repeat domain; EFhs, EF hands; GAR, GAS2-related domain; GSRs, Gly-Ser-Arg repeats; EBBS, EB1/EB3-binding site containing a Ser-X-Ile-Pro motif (where is X is any residue); MTBD, microtubule-binding domain. BPAG1a (NP_598594) is 5379 res. long. BPAG1b-specific domain (2014 res. long, deduced from NP_604443; 7393 res.) is inserted in between SR10 and SR11 of BPAG1a (after res. 1548).

The BPAG1a/b isoforms are made up of multiple modular domains. They possess an actin-binding domain (ABD) and a plakin domain in their N-terminus, and an MT-binding domain (MTBD) in their C-terminus (Fig. 1). The latter is composed of a growth arrest-specific protein 2 related (GAR) domain, which binds to and stabilizes MTs and a glycine-serine-arginine (GSR) repeat-containing region, which bundles MTs [10]. In addition, the C-terminal extremity of BPAG1a/b is able to form a complex with end-binding protein 1 (EB1) [11]. EB1 is a core component of the MT plus end complexes, which autonomously tracks MT plus ends and recruits other proteins. Furthermore, BPAG1a is a binding partner of p150^Glued^ subunit of dynactin [12], which also interacts with MT plus end proteins. Dynactin is thought to mediate the binding of dynein to cargos such as membranous organelles [13]. Interestingly, BPAG1a is also a binding partner of endocytic vesicle proteins called transmembrane protein 108 (or retrolinkin) and clathrin [14], [15].

The importance of the various BPAG1 isoforms is best attested by the dramatic consequences observed in cases of genetic defects of BPAG1. Naturally occurring mutations as well as engineered inactivation of *Dst* in mice cause *dystonia musculorum (dt)*, a disease characterized by sensory and motor neuron degeneration, and early death [16]–[18]. In humans, a pathogenic mutation affecting the MTBD of BPAG1a/b results in sensory autonomic neuropathy with dysautonomia, severe psychomotor retardation, and early death [19], while disruption of BPAG1a/b due to a chromosome breakpoint in the middle of one of the *DST* gene copies, is associated with encephalopathy, motor and mental retardation, and visual impairment [20]. *Dst*-null mice showed also discrete signs of skin blistering as a result of an impaired attachment of keratin IFs to hemidesmosomes in basal keratinocytes due to the absence of BPAG1e [21]. In humans, by analogy, homozygous nonsense *DST* mutations affecting BPAG1e result in epidermolysis bullosa simplex with fragility of basal keratinocytes and skin blistering [22], [23].

In skeletal muscle and cardiac tissues, BPAG1b is found colocalized with Z-discs, intercalated discs, and sarcolemma, but not with myosin and, surprisingly, actin [24], [25]. Furthermore, *dt* mice exhibit an intrinsic muscle weakness, increased muscle fatigability and sarcolemmal fragility, and an altered myotube cytoarchitecture [26], suggesting that BPAG1b has important roles in muscles.

In this study we sought to gain better insight into the complexity of BPAG1 isoforms and their role in MT organization and stabilization in the mouse myoblast cell line C2.7. We have identified novel mouse BPAG1a/b (and MACF1a/b) isoforms due to alternative splicing of the 3′ end of their pre-mRNA affecting the C-tail of the proteins. By using siRNA-mediated *Dst* silencing, we further characterized the impact of BPAG1 isoforms on MT stability, cytoskeletal organization, cell migration, vesicular transport and cell adhesion of C2.7 myoblasts.

## Results and Discussion

### Novel variants of BPAG1a and/or b and MACF1a and/or b and their tissue expression profile

**Figure 2:**
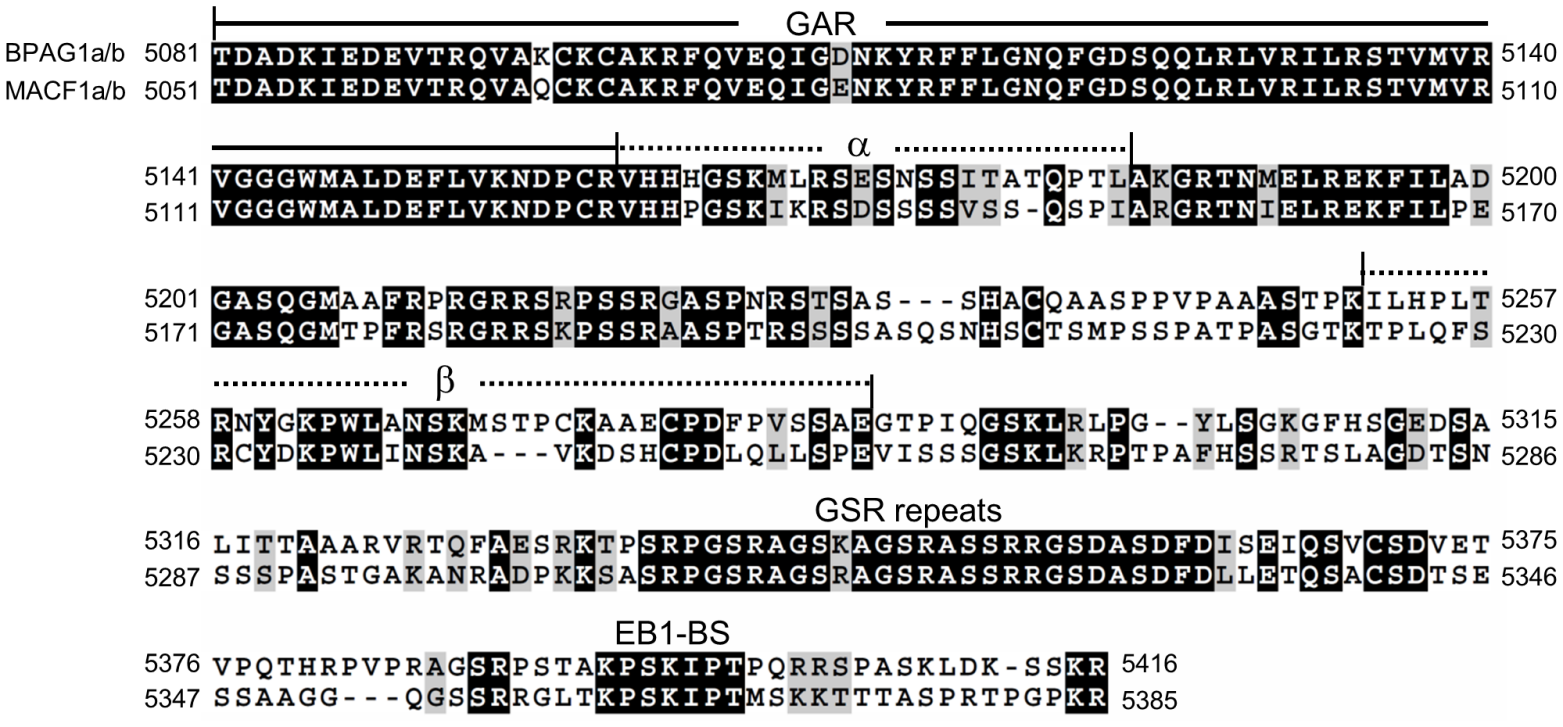
Protein sequence alignment of the C-terminus of BPAG1a/b and MACF1a/b. Identical amino acids are shaded in black with white letters. Conserved amino acids changes are shaded in grey. The GAR domain, sequences encoded by the alternatively spliced exons α and β, GSR repeats and EB1-binding site (EB1-BS) are indicated. The denomination «C-tail» corresponds to the sequence(s) after the GAR domain.

Three different transcription initiation sites can result in the expression of three BPAG1a and/or b isoforms with different N-terminal sequences. These variations either precede the ABD in isoforms 1 and 2, or change the structure of the ABD in isoform 3, affecting the actin-binding activity of the proteins [4], [27]–[29]. By analogy with the N-terminus, we investigated the existence of different isoforms with various C-terminal sequences that interact with MTs. By using the 3′ genomic region of the mouse *Dst* gene to perform a BLAST search in the mouse expressed sequence tag (EST) database, we found several EST clones. Comparison of these ESTs to the reported cDNA sequences of BPAG1a/b revealed one additional unreported sequence (exon termed β) in between the third and second to the last exon of each gene as well as the alternative use of an upstream exon (exon α) (Fig. S1). Two major isoforms of MACF1, a and b, have sequences similar to those of BPAG1a/b. Alternative splicing at the 3′ end of *Macf1* pre-mRNAs similar to that of *Dst* was also found (Fig. S1). Therefore, alternative splicing potentially yields four C-terminal BPAG1a and/or b (and MACF1a and/or b) isoforms termed, nBPAG1, αBPAG1, βBPAG1, and αβBPAG1 (with “n” denoting the absence of amino acid stretches encoded by exons α and β) (Fig. 2 and S2A).

**Figure 3:**
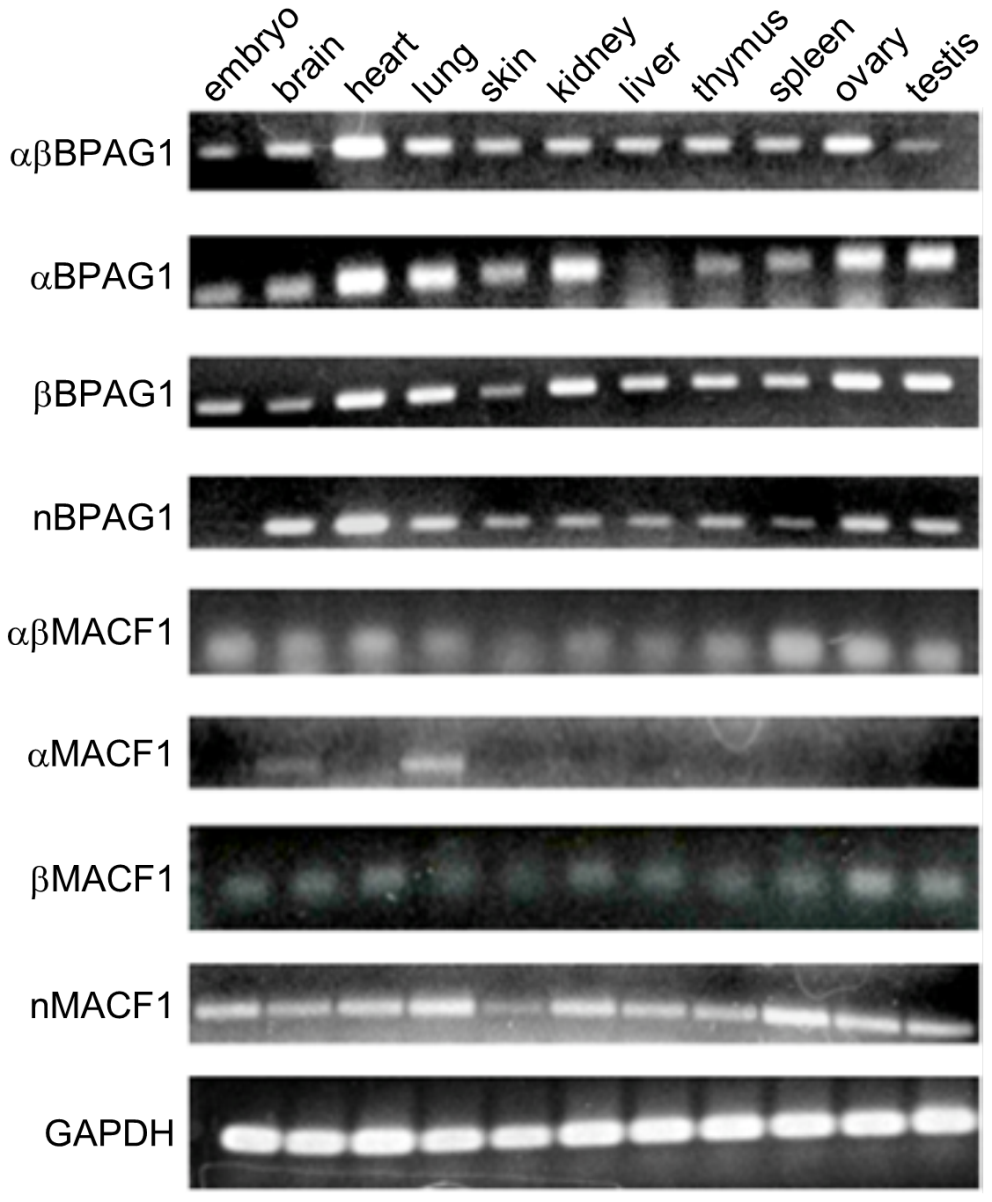
Expression profile of BPAG1a/b and MACF1a/b 3′-variants in various mouse tissues. RT-PCRs were performed with primers specific to the various plakin variants or to GAPDH from total RNA. The amplicons were analyzed on agarose gel and stained with ethidium bromide.

To determine if the EST sequences were indeed part of the coding regions of *Dst* and *Macf1* genes, we performed RT-PCR studies using mouse brain and heart RNAs. Primers were designed for the already documented and newly discovered 3′ ends (listed in Table S1). The sequences of the amplified cDNA fragments were identical to those predicted for the novel variants. Next, we performed RT-PCR using variant-specific primers against total RNA isolated from different mouse organs (Fig. 3). The results showed that most variants are ubiquitously expressed, except for αMACF1 mRNA, which is only detected in brain and lung. Nevertheless, we observed some variant-specific differences in the expression profile. Specifically, we did not detect the expression of nBPAG1 in embryonic tissue in contrast to all the other BPAG1 variants, whereas αBPAG1 is absent from the liver.

### All the different BPAG1a/b C-terminal isoforms can bundle MTs and interact with EB1 and EB3

The MTBD is composed of a GAR domain that co-localizes with and stabilizes MTs and a GSR-repeat domain that is able to bundle MTs in transfected COS-7 cells [10]. The alternative splicing described above affects sequences within the C-tail of BPAG1a/b in between the GAR domain and the GSR repeats (Fig. 2). Overexpression of all BPAG1 C-tail constructs induced MT bundling in transfected COS-7 cells (Fig. S2B). Similar results were also obtained for MACF1 C-tail constructs (Fig. S2C), indicating that the bundling activity of the GSR-repeat region is not significantly affected by flanking sequences.

Previous studies showed that by means of sequences containing two SxIP motifs the invertebrate spectraplakin Shot is able to recruit EB1 and adenomatous polyposis coli (APC) as well as to promote MT assembly at the muscle-tendon junction and in neurons [9], [30]. Positively charged residues in Shot C-tail also contribute to its interaction with EB1 and act as an MT stabilizer [9]. BPAG1a/b and MACF1a/b contain a single SxIP motif in their C-tail (Fig. 2). This motif is important for their interaction with the C-terminal end of EB1 [11], [31]–[33] (for a review, see [34]). BPAG1b was previously shown to co-localize with GFP-EB3 in HFFF2 fibroblasts [35]. EB3 is a neuronally expressed EB family member which is also upregulated in muscle cells upon differentiation [36]. We found that glutathione-agarose beads charged with GST-EB3-C could pull down all FLAG-tagged BPAG1-C-tail isoforms (Fig. S3) or nMACF1-C-T (data not shown) from transfected COS-7 cell extracts. Similar results were obtained with GST-EB1-C (data not shown). Our results thus indicate that various C-terminal BPAG1a/b isoforms are able to form a complex with the two end-binding family members, EB1 or EB3. Knockdown of EB1 or EB3 prevents elongation and fusion of myoblasts into myotubes [36], [37]. Since BPAG1a/b interact with both EB1 and EB3, it is plausible that BPAG1a/b are effectors of their biological functions.

### BPAG1a/b show a complex staining pattern in C2.7 myoblasts

**Figure 4:**
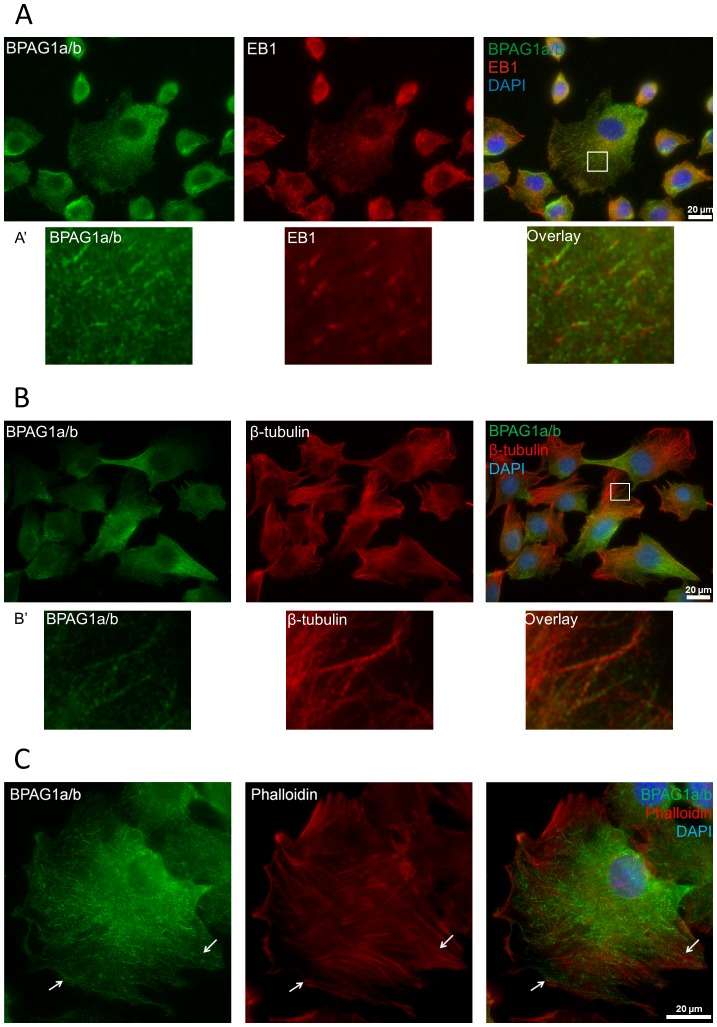
Localization of BPAG1a/b in C2.7 myoblasts. Fixed cells were costained with anti-BPAG1a/b serum R18024 (green) and A) anti-EB1 antibody, B) anti-β-tubulin antibody, or C) TRITC-phalloidin (all in red). Blue: nuclei stained with DAPI. Scale bar: 20 µm. BPAG1a/b have a dot-like and short-filament-like pattern. Short filaments of BPAG1a/b co-localize with EB1 comets (see A′), microtubules (see B′), and locally co-align with actin fibers (white arrows). Note in panel B the opposite intracellular abundance of BPAG1a/b *versus* MTs. White frames indicate areas magnified in A′ and B′.

Since in the above studies, we were unable to identify a functional difference among the new BPAG1a/b isoforms, we next investigated the role of BPAG1a/b in myoblasts using the C2.7 myoblast cell line, in which both BPAG1b and, less abundantly, BPAG1a isoforms are expressed [24], [26]. All isoforms were knocked down using two distinct siRNAs targeting sequences within the plakin domain of BPAG1 (here referred to as BPAG1 siRNA1 and siRNA3, respectively). Efficiencies of siRNA-mediated knockdown were quantified by immunoblotting (Fig. S4A). Using both BPAG1 siRNAs, we observed an approximately 90% knockdown efficiency in several independent experiments as assessed at 24 h and 48 h after transfection (Fig. S4B). We verified the specificity of the immunofluorescence signal obtained using the rabbit antiserum R18024 directed against BPAG1a/b in C2.7 myoblasts after siRNA-mediated silencing of all known BPAG1 isoforms. In non-transfected C2.7 myoblasts or those transfected with control siRNA, the R18024 antiserum showed a staining pattern characterized by cytoplasmic bright dots and filamentous structures of various lengths. The bright dots were usually asymmetrically distributed in the cytoplasm, in perinuclear locations as well as at the cell periphery (Fig. 4 and Fig. S4C). The short filamentous pattern was especially well appreciated in large lamellipodia (Fig. S4C, arrowheads). These staining patterns were almost completely absent in cells treated with either BPAG1 siRNA1 (Fig. S4C) or siRNA3, confirming the specificity of the signals obtained using the antiserum R18024.

**Figure 5:**
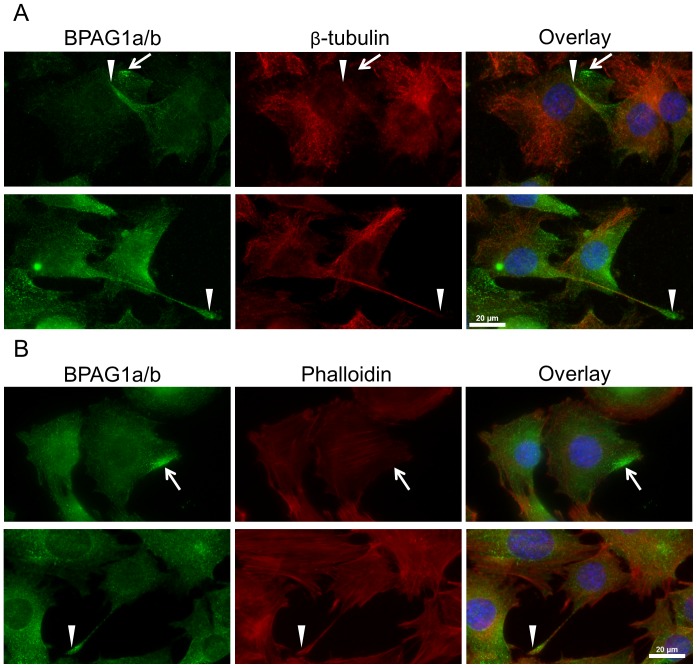
BPAG1a/b is enriched in pseudopodia and small areas at the plasma membrane. C2.7 cells were grown to high confluency, fixed in PFA, and stained with anti-BPAG1a/b (green) and A) anti-β-tubulin (red) antibodies or B) TRITC-phalloidin (red). Blue: nuclei stained with DAPI. Scale bar: 20 µm. Arrows indicate strong BPAG1a/b signals close to the plasma membrane and arrowheads BPAG1a/b localization in pseudopodia.

Pseudopodia are implicated in myoblast adhesion and fusion with neighboring skeletal muscle cells [38]. When we examined BPAG1a/b pattern in confluent C2.7 cells, long thin protrusions corresponding to pseudopodia were observed (Fig. 5, arrowheads). These structures, which contained actin but lacked MTs at their tips, displayed a peculiar intense BPAG1a/b staining (Fig. 5). Since there were additional sites close to the cell membrane of C2.7 cells showing high BPAG1a/b staining intensity (Fig. 5, arrows), which were reminiscent of nascent pseudopodia, it is conceivable that BPAG1a/b play a role in the formation and function of pseudopodia in mononucleated skeletal muscle cells.

### BPAG1a/b filaments mainly co-distribute with MTs

In double immunofluorescence microscopy studies of C2.7 myoblasts, BPAG1a/b showed a short filamentous staining, which partly overlapped with EB1. The latter displayed a characteristic comet-like staining (Fig. 4A) [37]. Interestingly, we observed that the region with the brightest EB1 signal at MT ends lacked BPAG1a/b signal, while a short stretch of co-localization was only noted in the tail of the EB1 comets (Fig. 4A, insets). The observed staining pattern was thus similar to that of Shot and DmEB1, the *Drosophila* orthologs of BPAG1/MACF1 and EB1, respectively [11]. When β-tubulin was co-stained, BPAG1a/b was found to localize along the MT lattice (Fig. 4B, insets). The polarized dot-like staining pattern of BPAG1a/b was most intense in areas where the MT signal was the weakest (Fig. 4B). Since BPAG1a/b filamentous structures did not invariably co-localize with all EB1 comet tails, BPAG1a/b likely associate only with a subset of the growing MTs. In transfected cells expressing FLAG-tagged, full-length BPAG1a, we observed short filament-like pattern and no asymmetric dot-like pattern. A truncated form of the protein that lacks all domains after the last spectrin repeat (FLAG-ABD-SR41) displayed a dot-like pattern homogenously spread throughout the cytoplasm (Fig. S5A and B). These observations indicate that the co-distribution of BPAG1a, and probably BPAG1b, with the MT network is affected by sequences contained in its C-terminal region.

Since BPAG1a/b have an ABD at the N-terminus [4], we next analyzed the relation of BPAG1a/b with the actin cytoskeleton. In most cells (Fig. 4C), there was no obvious co-localization between BPAG1a/b and actin stress fibers stained with phalloidin, although some short filamentous structures of BPAG1a/b were found co-aligned with actin fibers within large lamellipodia of some C2.7 cells (arrows in Fig. 4C). These results suggest that BPAG1a/b predominantly associate with the MTs but under certain conditions or according to their subcellular location are able to co-distribute with the actin cytoskeleton. Previous studies have shown that intramolecular interaction between ABD and MTBD of Shot prevents its interaction with actin [39]. A similar mechanism may thus also affect the topogenic fate of BPAG1a/b.

In line with our findings, Young *et al*. observed a strong perinuclear staining in C2C12 myoblasts using an antiserum directed against a peptide sequence specific to the N-terminal portion of the BPAG1a2/b2 isoforms [29]. These data imply that BPAG1a2/b2 specifically contribute to the perinuclear staining that we observed in C2.7 myoblasts using R18024 antiserum. In contrast to our data, Young *et al.* also described co-localization of BPAG1a2/b2 with actin stress fibers in the cell center of 5-10% of C2C12 myoblasts as well as a nuclear staining of BPAG1a2/b2 in all cells [29]. Since the R18024 antiserum used in our study binds to the SR region and should hence recognize all N-terminal isoforms of BPAG1a/b [40], the reasons for these staining differences remain unclear. Finally, in contrast to MACF1a [8], which co-localizes with focal adhesions (FAs) in keratinocytes, BPAG1a/b did not co-localize with vinculin in FAs of C2.7 cells (Fig. S6A).

### Integrity of MT network is important for the filamentous pattern of BPAG1a/b

**Figure 6:**
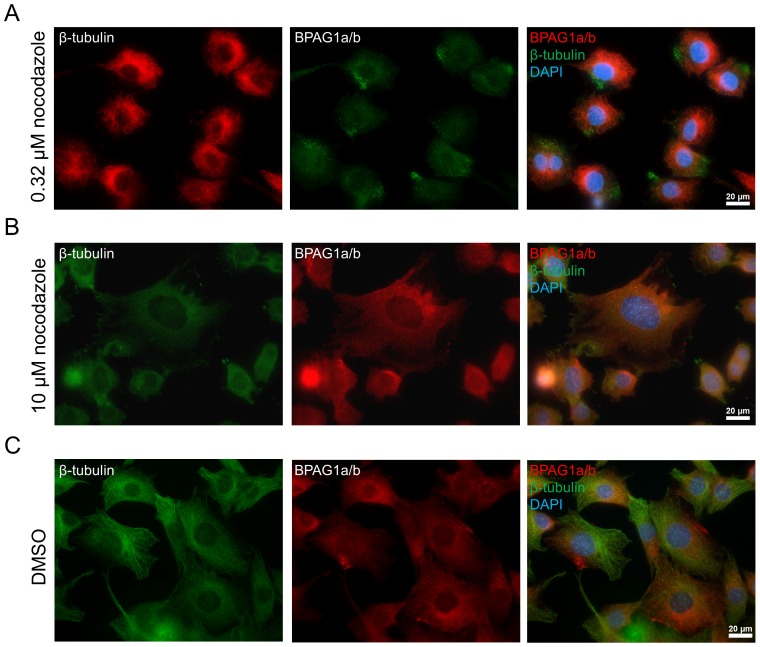
BPAG1a/b short filament pattern disappears upon microtubule depolymerization, but not the dotted pattern in C2.7 myoblasts. Cells were treated for 10 min with A) 0.32 µM or B) 10 µM nocodazole, then immediately fixed in PFA and immunolabeled with anti-β-tubulin and anti-BPAG1a/b antibodies. Blue: nuclei stained with DAPI. Scale bar: 20 µm. C) Negative control cells were treated with a corresponding volume of DMSO for 30 min, and processed for microscopy the same way. DMSO had no effect on the cells and BPAG1a/b pattern looked the same as in non-treated cells (Fig. 4B).

To analyze the impact of MTs on the subcellular distribution of BPAG1a/b, C2.7 myoblasts were treated with either nocodazole, an MT-depolymerizing drug, or taxol, an MT-stabilizing drug. Using two different nocodazole concentrations (10 µM and 0.3 µM), the MT network was either completely or partially disrupted. In the latter case, the MTs were very short and curled. In both tested conditions, the short filament-like pattern of BPAG1a/b disappeared, consistent with the idea that the filamentous staining pattern of BPAG1a/b is dependent on intact MTs. In contrast, the polarized dot-like staining pattern persisted upon nocodazole treatment (Fig. 6). Since the latter dot-like pattern was also found in cells not treated with nocodazole, particularly in areas where the MT staining signal was the weakest (Fig. 4B), it is very likely that this is a pool of BPAG1a/b, which is not associated with MTs.

Taxol but not DMSO treatment of C2.7 cell resulted in the disappearance of the comet-like staining pattern of EB1, usually leaving only faint and delicate filamentous structures as described before [41]. In contrast, BPAG1a/b pattern generally appeared unaffected, although some cells displayed a pattern of somewhat longer BPAG1a/b filaments. Finally, EB1 overlap with BPAG1a/b increased, especially in areas of high BPAG1a/b density, suggesting that EB1 interacts more strongly with BPAG1a/b when MTs are stabilized (data not shown).

### BPAG1a/b are not essential for the cytoskeleton organization of MTs and actin stress fibers

Shot is important for the stability of MTs in neuronal axons. In the axons of Shot-null mutant neurons, nocodazole treatment results in extensive areas devoid of MTs, which are not observed in wild-type neurons [9]. MACF1a is also implicated in MT organization in neurons [42]. Based on these findings, Shot, MACF1a, and other spectraplakins are thought to be functionally related to MT-associated proteins (MAPs), acting as MT-stabilizing factors [9]. By analogy, we next assessed the organization of the MT network and EB1 pattern in BPAG1-knockdown cells, but were unable to detect any significant change by immunofluorescence microscopy (data not shown). Furthermore, when C2.7 cells were subjected to cold-induced MT depolymerisation, the MT network seemed to be affected to a similar extent in both control and BPAG1 knockdown C2.7 cells as analysed by immunofluorescence studies as well as by immunoblotting of the soluble and insoluble MT fractions (data not shown). Finally, as assessed by immunofluorescence, there was no difference in the depolymerisation of the MT network between control and BPAG1 knockdown C2.7 cells upon treatment with 0.3 µM and 10 µM nocodazole nor in the repolymerization of MTs after washing out nocodazole (10 µM, data not shown). Our findings are in line with previous studies showing that knockdown of BPAG1 in HFFF2 fibroblasts does not lead to any major change in the dynamics or organization of MTs [35]. Since myoblasts and fibroblasts are structurally similar, and fibroblast conversion to myogenic cells has been reported [43], it is very unlikely that BPAG1a/b serve as MT stabilizing proteins in these cell types.

Finally, by immunofluorescence analysis, we examined the staining pattern of the actin network in BPAG1-knockdown myoblasts using phalloidin. When compared to control cells, BPAG1 knockdown had no impact on the MF network in transfected cells (data not shown). Therefore, BPAG1a/b do not seem essential for either the stability of MTs or the organization of MT and MF networks in C2.7 myoblasts.

### BPAG1a/b and vesicular transport

**Figure 7:**
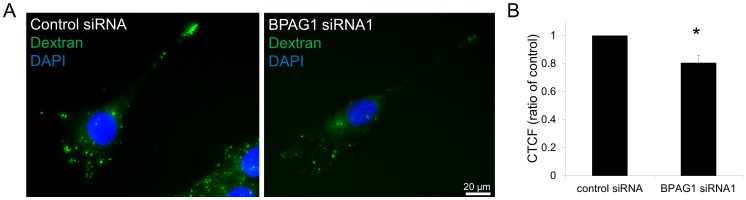
BPAG1 knockdown leads to a reduction in dextran uptake and/or retention within the C2.7 myoblasts. A) Sparsely seeded cells were incubated with FITC-dextran (green) for 10 min and then chased for 1 h. Afterward cells were fixed in PFA, stained with DAPI (blue), and mounted. Scale bar: 20 µm. B) Graph represents quantification of FITC signal that was measured using ImageJ for each individual cell (84–104 cells/condition). CTCF (corrected total cell fluorescence)  =  Integrated Density – (Area of selected cell × Mean fluorescence of background readings). Data are mean ± SEM, *p = 0.03 (n = 3 independent experiments).

BPAG1a directly interacts with several vesicle-associated proteins such as dynactin components, transmembrane protein 108, and clathrin [12], [14], [15]. In neuronal cells, BPAG1a was found to co-localize with vesicles along MTs [12]. We thus analysed FITC-dextran uptake by control and BPAG1-knockdown C2.7 cells using immunofluorescence microscopy. Three independent experiments consistently showed an average reduction of 20% of the dextran signal intensity in BPAG1-knockdown cells (Fig. 7). This observation argues for some perturbation in the endocytic pathway responsible for either a lower dextran uptake and/or an increased recycling of the dextran-containing vesicles back to the plasma membrane.

To dissect potential mechanisms responsible for these latter results, we analysed the relation of BPAG1a/b with both early and late endosomal markers by expressing GFP-tagged Rab5 and Rab7, respectively. Immunofluorescence microscopy analysis in C2.7 myoblasts revealed no obvious co-localization between BPAG1a/b and either Rab5 or Rab7. Furthermore, BPAG1 knockdown did not alter the staining pattern of Rab5 and Rab7 (data not shown), an observation implying that BPAG1a/b are associated with a different and less abundant type of vesicles. The latter may explain why BPAG1 knockdown resulted in only a moderate decrease of the dextran signal in C2.7 cells.

In a recent study, BPAG1 has been shown to interact and to co-localize with herpes simplex virus (HSV) capsids in HFFF2 fibroblasts. Furthermore, inactivation of BPAG1 in these cells negatively affected the movement of HSV capsids in anterograde and retrograde manner [35], [44]. In combination with our data, these results suggest that BPAG1 is involved in cargo transport along the MTs rather than in maintenance of MT structure and organization in these cells.

### BPAG1a/b role in maintenance of Golgi apparatus structure

**Figure 8:**
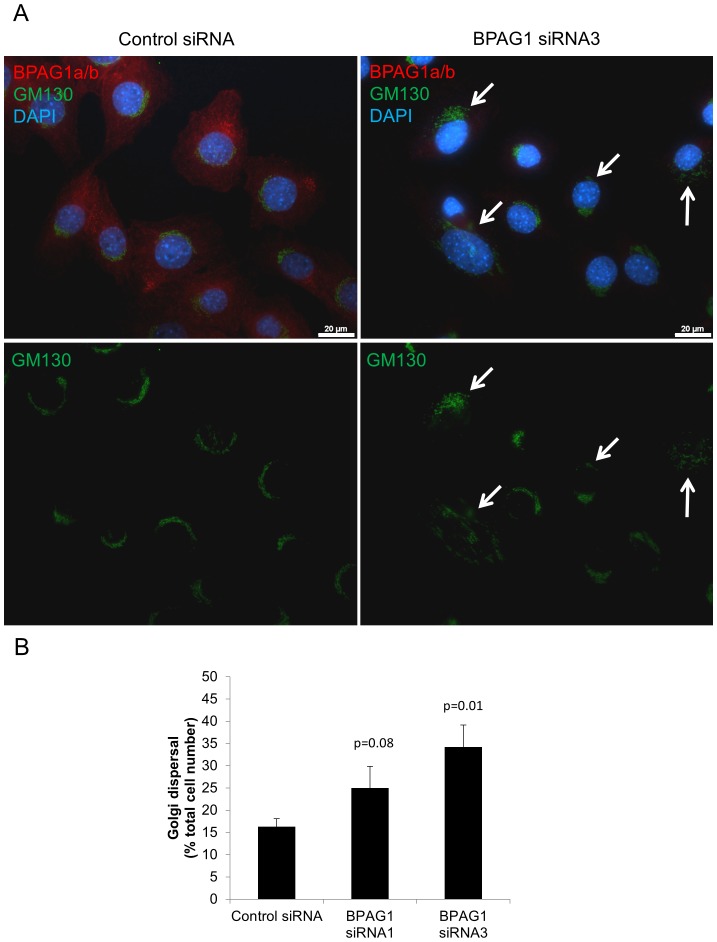
Knockdown of BPAG1a/b in C2.7 cells induces the dispersal of the Golgi apparatus. A) Cells treated with control and BPAG1 siRNA were fixed in PFA and immunolabeled with anti-GM130 (cis-Golgi marker, green) and anti-BPAG1a/b (red) antibodies. Blue: nuclei stained with DAPI. Scale bar: 20 µm. Arrows: examples of dispersed Golgi. B) Graph represents percentage of cells (200 cells per siRNA) with dispersed Golgi. Data are mean ± SEM, n = 3 independent experiments.

BPAG1 knockout in primary neurons and knockdown in HEK 293T cells results in an increased dispersal of the Golgi apparatus [45]. We therefore analysed Golgi structure in C2.7 myoblasts using GM130, a marker of the *cis*-Golgi. As assessed by immunofluorescence, BPAG1 knockdown also led to an increased Golgi dispersal in C2.7 cells (Fig. 8). The Golgi dispersal induced by BPAG1 knockdown was not increased by pre-treatment of the cells with brefeldin A to induce complete disassembly of Golgi, followed by a washout (data not shown). Since no obvious co-localization between BPAG1a/b and GM130 was found, the effect of BPAG1 on the Golgi apparatus structure is probably indirect, although the presence of a minor fraction of BPAG1a/b associated with the Golgi cannot be excluded. Please note that figure 8A also demonstrates BPAG1 knockdown efficiency by siRNA3 and sites of high BPAG1a/b staining intensity close to the cell membrane.

The increased Golgi dispersal observed in BPAG1-knockdown neurons has been related to the ability of BPAG1 to associate with MAP1B by means of its plakin domain [15]. The latter was reported to be required for MAP1B recruitment to centrosomes. MAP1B promotes acetylation and stabilization of MTs, which is important for maintenance of the Golgi structure at the centrosome [45]. The Golgi apparatus is also dependent on dynein and dynactin for its localization at the centrosome [46], [47]. To assess whether the plakin domain and its binding to MAP1B is critical for the effect of BPAG1a/b on Golgi structure, we expressed in C2.7 cells two BPAG1a/b recombinant proteins encompassing the spectrin repeat 21 (SR21) through the GAR domain fused to mCherry (mCherry-SR21-GAR) and SR21 through SR41 (mCherry-SR21-SR41, Fig. S5A). Both of these recombinant proteins contain sequences shown to bind to p150^Glued^ [12], but lack the plakin domain. In contrast to what was observed in BPAG1-knockdown C2.7 cells, the Golgi showed a more compact structure in around 70 % of transfected cells expressing mCherry-BPAG1a/b fusion constructs when compared to cells transfected with the control mCherry construct (37 %) or non-transfected cells (18 %) (Fig. S7). A similar trend was observed in C2.7 cells transfected with FLAG-tagged, full length BPAG1a and FLAG-ABD-SR41 in comparison to FLAG-PRD2. However, since only few transfected cells expressed these two large BPAG1a/b recombinants, no statistical analysis was possible. Our results indicate that sequences contained within the SRs of BPAG1a/b are responsible for the regulation of the Golgi structure by BPAG1a/b and suggest that these effects are mediated via its interaction with p150^Glued^.

### BPAG1 plays a role in directional C2.7 myoblast migration

**Figure 9:**
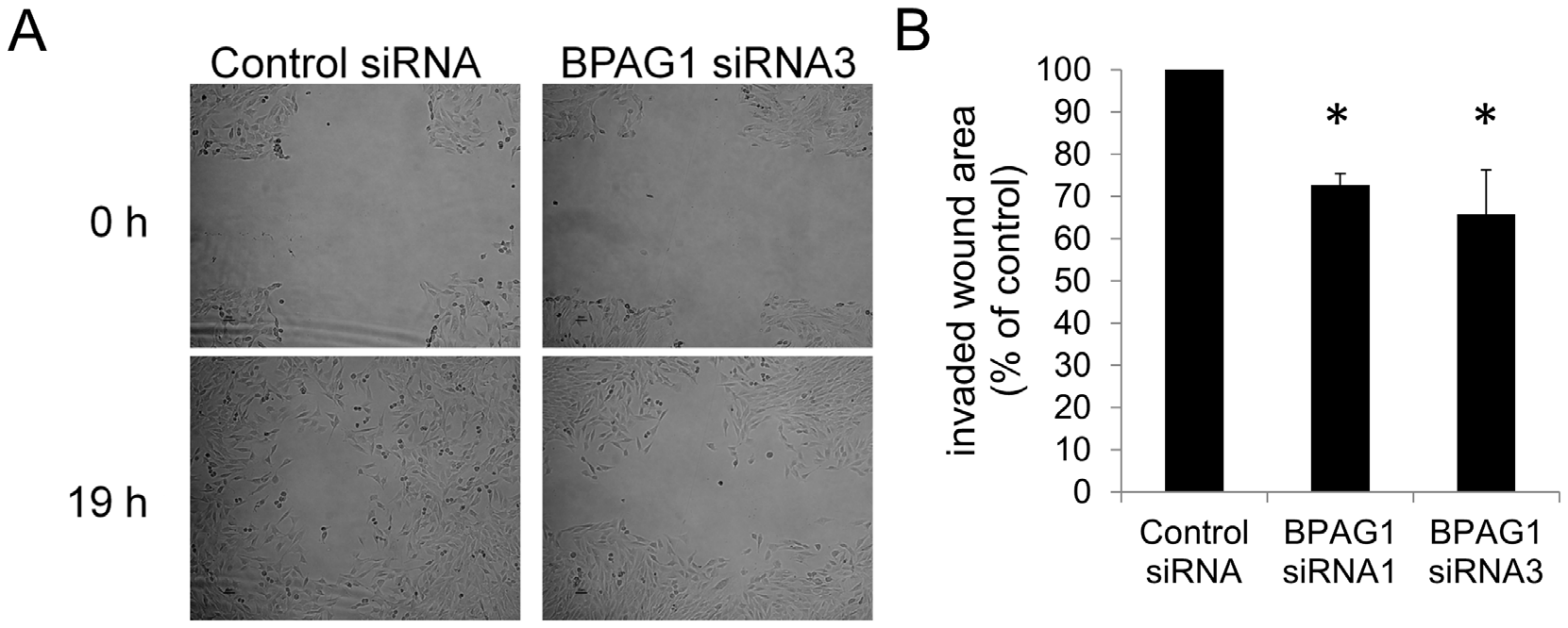
BPAG1 knockdown reduces C2.7 myoblast wound invasion in a wound healing assay. A) Pictures of denuded areas in C2.7 cell layer were taken immediately after the scratch was applied (0 h) and 19 h later. Images are representative of three independent experiments. Scale bar: 50 µm. B) Graph represents quantification of wound areas reinvaded by cells by subtracting wound area at 19 h from wound area at 0 h. Results were expressed in comparison to myoblasts transfected with control siRNA. Data are mean ± SEM. *p<0.05 *versus* control, Student's t test, n = 3 independent experiments, 3 crosses per triplicate for each siRNA.

Both BPAG1e and MACF1a are important for cell adhesion and migration [8], [21], [48]. These processes are relevant for skeletal muscle repair and regeneration, since myoblasts need to migrate towards damaged myotubes, adhere to and fuse with them [49]. Vesicular transport, which is affected by BPAG1a/b as observed in this study, is another important process for cell migration [50]. Therefore, to test the role of BPAG1a/b in myoblast migration, we carried out a wound healing assay in BPAG1-knockdown C2.7 cells. In three independent experiments, BPAG1-knockdown C2.7 cells showed an average 27% (siRNA1) and 34.3% (siRNA3) lower wound invasion compared to control siRNA-treated cells (Fig. 9).

**Figure 10:**
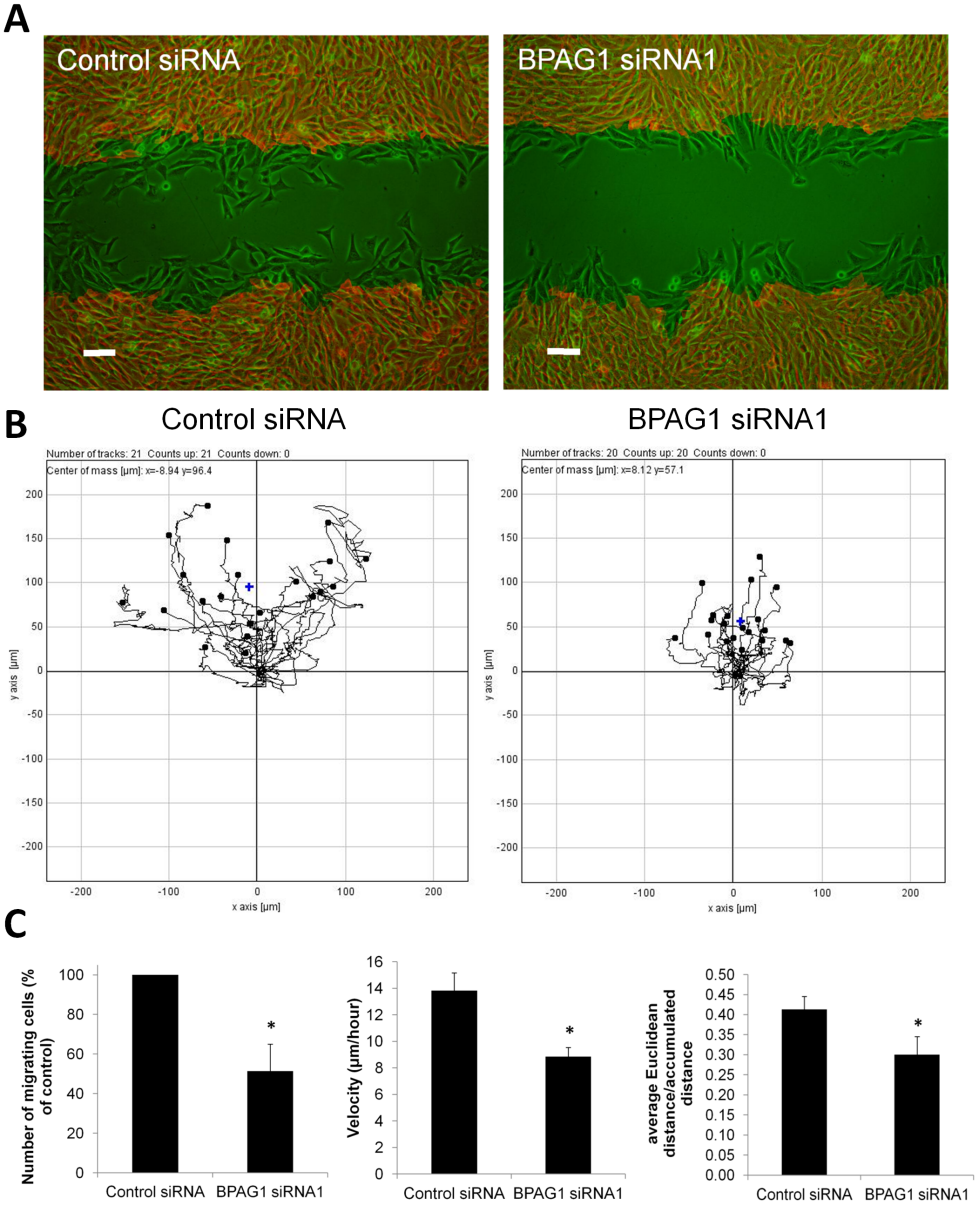
BPAG1 knockdown reduces the number of myoblasts migrating into a wound, myoblast migration velocity and directness in a wound healing assay. A) The fields of view at 0 h (red) and at 18 h (green) after scratch were overlaid to visualize the invading cells. Cell-free area at 0 h was colored in black to increase contrast on the overlay. Scale bar: 100 µm. B) Time lapse photographs were taken of control and BPAG1 knockdown myoblasts every 10 min for 18 h. The migratory paths of individual cells that did not divide during 18 h are shown from one of the three experiments from the lower wound edge (see panel A). C) Quantitative comparison of the number of cells transfected with BPAG1 siRNA that had migrated into an acellular area with control siRNA transfected cells. The mean cell velocities were measured by manual tracking using ImageJ and pooled from three independent experiments. The mean velocity of BPAG1 knockdown cells is reduced by 36 %. Cell migration directness was quantified by calculating Euclidian distance divided by the accumulated distance for each individual cell (120 cells per siRNA). Data are mean ± SEM. *p<0.05 vs control, Student's t-test, n = 3 independent experiments.

To further investigate the migration defect of BPAG1-knockdown myoblasts, we used time-lapse microscopy to track migrating cells. This technique allowed us to focus on a specific region of the wound and to quantify the number of cells that migrate into the wound more accurately (Fig. 10A). Three independent experiments revealed an approximately 50% reduction in the number of cells that migrated into the wound (Fig. 10C). By cell tracking, we found a 36% reduction in cell velocity and 29% reduction in directness of cell migration (Fig. 10B and C). Cell counting revealed no significant differences in cell proliferation or death rate after knockdown of BPAG1 in C2.7 cells (data not shown), allowing an 19 h time course for the wound closure experiments. In line with our findings, primary myoblasts from *dt* mice were also found to have a normal proliferative potential [51].

In this context, we examined the BPAG1a/b staining pattern in migrating C2.7 cells at the wound edge, which were fixed 7 h after scratching. By immunolabeling with the R18024 antiserum, the most intense staining of BPAG1a/b was almost invariably observed in the trailing portion of migrating cells, which displayed a dotted and short filament-like pattern (Fig. S8). A similar polarized pattern of BPAG1a/b was also observed in myoblasts under sub-confluent conditions (Fig. 4 and Fig. S4C). Although the mechanisms responsible for this peculiar polarized distribution of BPAG1a/b remain to be dissected, our observations suggest that BPAG1a/b either exert a specific function in the trailing edge or change their subcellular redistribution secondary to post-translational modifications of MTs [52].

Time lapse microscopy studies with sparsely seeded C2.7 cells on cell culture plastic dishes indicated that BPAG1 knockdown reduced migration directness but did not impair the velocity of cell migration in these conditions (Fig. S9). Since the Golgi apparatus influences polarized vesicular trafficking and MT orientation toward the leading edge of the cell, its integrity is important for directional cell migration [53]–[56]. Therefore, it is very likely that decreased directness of migration of C2.7 myoblasts is the result of the increased Golgi dispersal observed in BPAG1-knockdown C2.7 cells (Fig. 8).

Cell-substratum adhesion is important for cell migration. Therefore, we tested the ability of BPAG1-knockdown C2.7 cells to adhere to plastic cell culture dishes, which were either non-coated or coated with collagen [36]. When compared to control cells, the number of knockdown cells that adhered to the coverslips in both tested conditions was similar (Fig. S6B and C). Since BPAG1a/b was not found to co-localize with vinculin in FAs, while the staining pattern and density of FAs were similar between control and BPAG1-knockdown C2.7 cells (Fig. S6A), a contribution of BPAG1 to initial myoblast adhesion seems very unlikely.

MACF1 deficiency impairs the migration of keratinocytes by reducing the targeting of MTs along F-actin to FAs and by stabilizing FA-actin networks. An intrinsic actin-regulated ATPase in MACF1 has been reported for these biological functions [8], [21], [48]. Despite their similar protein sequences, MACF1a/b and BPAG1a/b do not seem to have the same function.

## Conclusions

Our study reveals that alternative splicing of two exons coding for the C-tail results in four additional variants of BPAG1a/b, which are expressed in various mouse tissues. These isoforms can all bundle MTs and interact with the MT plus end proteins EB1 and EB3. Our results show that the filamentous pattern of BPAG1a/b in C2.7 myoblasts is due to its association with MTs, whereas the reason for the dot-like pattern remains unclear. Knockdown studies in C2.7 myoblasts show that BPAG1a/b have no effect on the organization of the MT and MF networks, but rather affect endocytosis and the Golgi apparatus structure. The latter is either dispersed upon knockdown of BPAG1 or more compact upon overexpression of BPAG1a/b SR regions that can bind p150^Glued^. Finally, knockdown of BPAG1 does not impair cell adhesion, but results in decreased directness of cell migration, for which Golgi dispersal may be responsible. Collectively, these results provide further insights into the complex functions of BPAG1a/b in the membrane-bound organelle transport along MTs, in maintenance of the Golgi apparatus structure as well as in migration of C2.7 myoblasts.

## Materials and Methods

### RT-PCR

Equal amounts of total RNA extracted from various mouse tissues (Clontech) were subject to one step RT-PCR (Qiagen). The primers used are listed in Table S1.

### Plasmid construction

The mouse αBPAG1a and MACF1a protein sequences GeneBank accession No: NP_598594 and NP_001186066 respectively were taken as references for labeling residue number. For cloning of the C-Tail of BPAG1 and MACF1 PCR was performed on mouse (BALB/c) brain QUICK-Clone cDNA (Clontech) using Advantage cDNA PCR kit (Clontech) with the primers listed in Table S1. Cycling parameters were set according to the manufacturer's protocol. PCR products were cloned into pCR2.1-TOPO vector (Invitrogen). cDNA clones were sequenced. The corresponding pTOPO clones described above were digested with EcoR1/EcoRV and cloned into pcDNA3.1-FLAG vector [57]. The FLAG-tagged constructs prepared for this study were the following: nBPAG1-C-tail (res. 5157–5355), α (res. 5157–5379), β (res. 5157–5392), and αβ (res. 5157–5416) (see Fig. S1). For cloning full length αBPAG1a cDNA, two overlapping αBPAG1a cDNA fragments were amplified from mouse brain cDNA library, cloned into pCR3.1-TOPO-BluntII (Invitrogen) and sequenced. The two complementary pieces were joined together thanks to the unique *Avr*II site in αBPAG1a sequence. CMV-FLAG sequence from pcDNA3.1-FLAG was cloned in frame with αBPAG1a coding sequence (res. 2 to 5379). This final vector was called pTCF (for TOPO vector, CMV promoter, and FLAG tag). pCTF-ABD-SR41 codes for FLAG-BPAG1a res. 2–4953. Mouse BPAG1b PRD2 cDNA fragment, coding for res. 1549 to 2206 (GeneBank accession No: NP_604443), was cloned into pcDNA3.1-FLAG. Two BPAG1a fragments were cloned into pmCherry-C (Clontech) to obtain the mCherry fusion recombinant proteins: mCherry-SR21-GAR (res. 2669–5263) and mCherry-SR21-SR41 (res. 2669–4953) (see Fig. S5A). Constructs encoding GFP-Rab5 and GFP-Rab7 were described elsewhere [58], [59].

### Antibodies

The following primary antibodies were used rabbit antiserum R18024 and R18611 [40], rabbit anti-skeletal myosin heavy chain IgG (Santa Cruz Biotechnology), mouse anti-EB1 (BD Biosciences), -β-tubulin (Thermo Scientific), -vinculin (VII-F9, [60]), -GM130 (BD biosciences), -FLAG-M2 antibody (Sigma-Aldrich), and rat anti-FLAG tag (Stratagene). Secondary antibodies used for immunofluorescence studies were highly cross-absorbed Alexa Fluor 488 and 568 anti-rabbit IgG, Alexa Fluor 488 and 568 anti-mouse, and Alexa Fluor 488 anti-rat, all from Invitrogen. For western blotting secondary antibodies were IR-Dye 800cw anti-rabbit IgG (LI-COR) and anti-mouse Alexa 680 (Invitrogen).

### Cell culture and transient transfections

COS-7 cells were grown and transfected as previously described [10]. C2.7 mouse myoblast cell line [61] was cultured in DMEM (Invitrogen) supplemented to 20% with newborn calf serum (NCS, Sigma-Aldrich), 100 U/ml penicillin and 10 µg/ml streptomycin (PS, Sigma-Aldrich) for proliferation, and in DMEM with 4% NCS and PS for differentiation at 37°C in 5% CO_2_ atmosphere. Chemical stocks were dissolved in DMSO according to the manufacturers' recommendations. Cells were treated with nocodazole (Sigma-Aldrich) and taxol (paclitaxel, Sigma-Aldrich) at 10 µM for 10 min. One day before transfection, C2.7 cells were seeded on glass coverslips at 2.5×10^4^ cells/cm^2^. C2.7 cells were transfected using Lipofectamine 2000 (Invitrogen) for most constructs. For large constructs, namely full-length FLAG-αBPAG1a and FLAG-ABD-SR41, TransIT (Mirus) and X-tremeGene HP (Roche) transfection reagents were used according to the providers' instructions.

### Immunofluorescence

Cells grown on glass coverslips were either fixed in ice-cold methanol at −20°C for 10 min or in 4% paraformaldehyde (PFA) in phosphate-buffered saline (PBS) for 15 min at room temperature. Afterward, the methanol-fixed cells were air-dried and rehydrated in PBS. PFA-fixed cells were permeabilized with 0.1% Triton X-100 in PBS for 3 min. Coverslips were blocked with 1% bovine serum albumin in PBS (B-PBS) for 15 min at room temperature. Cells were labelled with primary antibodies diluted in B-PBS for 2 h, washed 3 times in B-PBS for 5 min each time, and then, incubated with secondary antibodies and TRITC-phalloidin or Alexa Fluor 488-phalloidin (Invitrogen) dissolved in B-PBS for 1 h. Then, the cells were washed one more time in B-PBS, 2 times in PBS, and incubated with DAPI nuclear stain for 5 min. Finally, the cells were mounted in fluorescent mounting medium (Dako). Fluorescence microscopy was performed using Eclipse 80i microscope (Nikon).

### Production of Glutathione-S-Transferase (GST) fusion proteins

Plasmids encoding mouse GST-EB1 (res. 1–268) and GST-EB1-C (res. 164–268) were a generous gift from Dr. Gregg Gundersen (Columbia University). Mouse EB3 and EB3-C sequences coding for res. 1–281 and 145–281, respectively were amplified from mouse brain cDNA by PCR using the primers listed in Table S1. *Escherichia coli* BL21 (DE3) strain was transformed with the GST-fusion constructs as well as pGEX-2T (empty vector) and recombinant protein expression was induced by IPTG according to standard methods. BL21 cells were then lysed by sonication in 20 mM Tris-HCl pH 7.5 containing 0.15 M NaCl, 1 mM EDTA, 1 mM PMSF, 10 µg/ml pepstatin, 10 µg/ml aprotinin, and 2 µg/ml leupeptin. Sarkosyl was added to lysates to a final concentration of 1.5%, and the lysates were gently mixed for 15 min. After centrifugation, supernatants were adjusted to 2% Triton X-100 and 1 mM CaCl_2_, and the GST-fusion proteins were charged on glutathione-agarose (Sigma-Aldrich). The beads were thoroughly washed with an excess of binding buffer.

### GST pull-down assay

COS-7 cells were transfected with FLAG-tagged: n, α, β, αβBPAG1-C-Tail, n, α, and αβMACF1-C-tail. After 48 h cells were lysed in ice-cold PBS containing 1% Triton X-100 and and 1% complete protease inhibitor cocktail (Roche). Soluble cell lysates were collected after centrifugation at 16,000×g for 10 minutes at 4°C and incubated with glutathione-agarose beads charged with GST-EB1, GST-EB1-C, GST-EB3, GST-EB3-C or GST for 2 h. The beads were collected, washed extensively and boiled in SDS loading buffer. Proteins were detected by SDS/PAGE and western blotting with anti-FLAG antibodies.

### BPAG1a/b knockdown

To knock down BPAG1, FlexiTube siRNA Mm_Dst_1 (BPAG1 siRNA1) or Mm_Dst_3 (BPAG1 siRNA3) (cat. # SI00984375 and SI00984389, Qiagen) was used. Control siRNA was from Microsynth (cat. # 1491991). C2.7 cells were transfected using Lipofectamine RNAiMAX (Invitrogen). For a 10 cm^2^ dish, 3 µl RNAiMAX and 1 µl siRNA (10 µM) were mixed in 500 µl DMEM and incubated at room temperature for 20 min. C2.7 cells were trypsinized and resuspended in proliferation medium without antibiotics (100 µl medium per 2.5×10^5^ cells). Cells and siRNA complexes were mixed, incubated for 5 min at room temperature, seeded at 2.5×10^4^ cells/cm^2^ and cultured in proliferation medium without antibiotics for 24 h.

### Preparation of cell lysates and Western blot analysis

COS-7 cell cultures were washed with phosphate buffered saline (PBS) and scraped in lysis buffer, consisting of ice-cold PBS containing 1% Triton X-100 and complete protease inhibitor cocktail (Roche). Soluble cell lysates were collected after centrifugation at 16,000×g for 10 minutes at 4°C. Proteins were separated by SDS–polyacrylamide gel electrophoresis (SDS–PAGE) and transferred onto Immobilon membrane (Millipore) using BioRad equipment. The blocked membrane was stepwise incubated with monoclonal FLAG-M2 antibody (Sigma-Aldrich) (1∶1,000) and with goat-anti-mouse IgG-Alexa Fluor 680 conjugate (1∶5,000 Invitrogen). For detection of BPAG1a/b by Western blotting, C2.7 cells were cultured in a 6-well plate for 24 h after being treated with siRNA as above. Cells were rinsed in PBS two times, lysed in buffer containing 62.5 mM Tris-HCl pH 6.8, 2.5% SDS, and 10% glycerol (100 µl/well of a 6-well plate) and scraped from the wells immediately. Then, the samples were heated for 3 min at 93°C and passed through 30 G syringe 3–5 times to reduce their viscosity due to DNA. 10 µl of the sample were used to determine protein concentration using DC protein assay (BioRad) according to provider's instruction. 150 µg of each sample were mixed with necessary amount of β-mercaptoethanol and bromophenol blue to bring to 5% and 0.002% final concentration respectively, and heated again as above. The proteins were separated in a 6% polyacrylamide SDS gel by electrophoresis at 100 V until the 175 kDa protein marker band (New England BioLabs) was at the bottom of the gel. The proteins were electrotransferred for 75 min at 100 V in precooled buffer with 25 mM Tris, 192 mM glycine, 0.1% SDS and 10% ethanol to nitrocellulose membrane (Carl Roth GmbH). The membrane was blocked in PBS with 0.1% Tween-20 (PBS-T) and 5% skimmed milk (M), incubated for 2 h with primary antibodies dissolved in PBS-T-M, washed 3 times in PBS-T-M, and incubated for 1 h with secondary antibodies dissolved in PBS-M. Finally, the membrane was rinsed once in PBS-M and twice in PBS. Protein bands were visualized and their intensity quantified using Odyssey infrared imaging system (LI-COR).

### Cell migration assay

Cells treated with control and BPAG1 siRNA 24 h before were trypsinized and seeded at 5% confluency into a 6-well dish pre-incubated overnight with proliferation medium because it contains vitronectin. This also made conditions more similar to the wound healing assay. The left-over cells were seeded on glass coverslips for control of knockdown efficiency by immunofluorescence. Cells in the 6-well dish were allowed to attach for 2 h and then switched to DMEM with 10 µg/ml of freshly dissolved mitomycin C (Applichem) for 3 h to inhibit cell division. Then, cells were switched to proliferation medium and imaged using BioStation CT (Nikon). Phase contrast images were taken every 10 min for 19 h with 4x lens at 3 different positions in each well. Velocity and directness of cell migration were measured by manual tracking and chemotaxis and migration plugin (Ibidi) in ImageJ.

### Wound healing assay

Cells were treated with control and BPAG1 siRNA for 24 h as above and cultured in a 12-well dish. Either one scratch per well or 3 parallel scratches and 1 scratch perpendicular to them to make 3 intersections (crosses) were applied to the cell monolayers using a 200 µl pipette tip. The medium was immediately changed to fresh proliferation medium to remove the detached cells. Then, a phase contrast picture was taken of each scratch cross immediately and 19 h later using Eclipse TE-2000-4 microscope (Nikon). Wound areas were measured using ImageJ. For time lapse microscopy, cells with one scratch per well were switched to fresh proliferation medium with 25 mM HEPES and placed under the Eclipse TE-2000-4 microscope equipped with a 37°C chamber, recording an image of the scratch every 10 min for 19 h. Velocity and directness of cell migration were measured by manual tracking and chemotaxis and migration plugin (Ibidi) in ImageJ.

### Ice-induced microtubule depolymerization assay

Protocol was mostly as described before [62]. Cells were switched to 4°C growth medium with 25 mM HEPES and incubated on ice for 0, 0.5, 1, 2, 4, 6 and 19 h. Vessels with ice and cells were kept at 4°C for all incubation times. Then, the cells grown on glass cover slips were permeabilized in BRB80 (80 mM PIPES pH 6.8, 1 mM MgCl_2_, 4 mM EGTA, 0.5% Triton X-100 for 30 sec prior to fixation and immunofluorescence analysis. The cells cultured in 6-well plates without cover slips were lysed in MT stabilization buffer (20 mM Tris, pH 6.8, 2 mM EGTA, 1 mM MgCl_2_, 0,5% NP-40, 140 mM NaCl, and 4.7 µM taxol) for 20 . Cells were scraped from the wells, transferred to 1.5 ml tubes, and centrifuged at 20,000×g. The supernatant (tubulin fraction) was transferred to fresh tubes. The pellets (MT fraction) were resuspended in 1x SDS sample buffer with 6 M Urea in 1/4th of the corresponding supernatant volume. Dissolved pellets were passed through 30 G syringe 3–5 times. 18.75 µl of each lysate (mixed with 6.25 µl 4x SDS sample buffer and heated at 95°C for 5 min) and 18.75 µl of dissolved pellet (not heated) samples processed for western blotting and densitometric analysis using Odyssey system (LI-COR).

### Dextran uptake assay

C2.7 cells were transfected with siRNA and seeded at a density of 1.25×10^4^ cells/cm^2^ on glass cover slips. 24 h later, the cover slips were either placed into PFA for KD control by immunofluorescence later, or transferred to a humidified, light-impermeable chamber. These cells were pulsed for 10 min with 5 mg/ml FITC-dextran (MW 10,000, Sigma-Aldrich) in pre-warmed proliferation medium. Then, the cells were quickly rinsed 3 times in warm proliferation medium, and chased for 1 h in the same medium. Finally, the cells were quickly washed in PBS once, fixed, stained with DAPI, and mounted.

### Cell adhesion

Procedure was mostly as described before [36]. C2.7 cells were transfected with siRNA as above and grown in proliferation medium for 24–48 h. Afterward, cells were trypsinized, counted, and seeded at 1000 cells/well into a 12-well dish coated with 50 µg/ml collagen (Invitrogen) in 0.02 M acetic acid overnight at 4°C, or at 35,000 cells/well into a non-coated 12-well cell culture dish pre-incubated with proliferation medium. Cells were allowed to attach for 70 min. Then, the unattached cells were washed away by rinsing the wells once with PBS. The attached cells were fixed in PFA, and stained with Giemsa solution (Sigma-Aldrich). The number of cells per mm^2^ was determined by overlaying a grid with 0.16 mm^2^ squares under the wells and counting the total number of cells attached.

## Supporting Information

Figure S1 **Genomic organization of the** ***Dst*** **and** ***Macf1*** **3′ exons.** The alternatively spliced exons are labeled α and β. The translated sequences are in blue, red, and grey.
(TIF)

Figure S2 **Recombinant BPAG1a/b-C-Tail isoforms and MACF1a/b-C-Tail isoforms bundle MTs in transiently transfected COS-7 cells.** A) Schematic representation of FLAG-tagged BPAG1a/b-C-Tail (T) proteins expressed in transfected COS-7 cells. The amino acid numbers, corresponding to the first residue encoded by each of the last four exons, as well as the C-terminal residue of BPAG1a, are indicated (see also Fig. 2). B) Cells expressing the indicated FLAG-BPAG1a/b-C-T isoforms were double labeled with polyclonal anti-FLAG antibody (A, D, G, J) and monoclonal anti-tubulin antibody (B, E, H, K). Ectopically expressed proteins showed a predominant nuclear staining and induced bundling of MTs even though co-localization of the different BPAG1a/b-C-T isoforms with bundled MTs was not systematically observed. C) same as B) for the indicated FLAG-MACF1a/b-C-T isoforms corresponding to the respective FLAG-BPAG1a/b-C-T isoforms (see Fig. 2 for MACF1a/b exon borders).
(TIF)

Figure S3 **BPAG1-C-T interacts with EB3 in GST pull-down assays.** COS-7 cells were transfected with different FLAG-tagged BPAG1-C-T constructs and their lysates were used in GST pull-down assays with GST, GST-EB3 (res. 1-281), or -EB3-C (res. 145-281) as indicated. The presence of FLAG-BPAG1-C-T associated with the glutathione-Sepharose beads was detected by Western blotting with an anti-FLAG antibody. GST-APC-C did not pull down FLAG-nBPAG1-C-T (data not shown).
(TIF)

Figure S4 **BPAG1a/b can be efficiently knocked down in C2.7 myoblasts.** A) Western blot analysis of BPAG1a/b and myosin heavy chain (MHC) levels in cells transfected with control (Ctrl) siRNA or BPAG1 siRNA1 or siRNA3. BPAG1a/b was detected with anti-serum R18611 and MHC was detected sequentially on the same blot. Results are representative of three independent experiments for 24 h after transfection and two for 48 h after transfection for BPAG1 siRNA1 and siRNA3. B) Quantification of BPAG1a/b knockdown by normalization to MHC. Data are mean ± SD. C) Cells treated with control siRNA or BPAG1 siRNA1 for 24 h were fixed in PFA and immunolabeled with anti-BPAG1a/b serum R18024. Scale bar: 20 µm. BPAG1a/b have a dot-like (arrows) and short-filament-like (arrowheads) pattern. The same results were obtained for cells treated with siRNA3 (data not shown, see also Fig. 8). D) The three types of BPAG1a/b pattern were quantified in cells not treated by siRNA (NT), treated with control siRNA, BPAG1 siRNA1 or siRNA3 (n = 204–235 cells/condition).
(TIF)

Figure S5 **FLAG-BPAG1a and FLAG-ABD-SR41 display strikingly different patterns.** A) BPAG1a constructs used to transfect C2.7 cells. The borders of the FLAG- and mCherry-tagged recombinant proteins are indicated by black lines under the scheme of αBPAG1a. B) Cells were transfected with the indicated constructs, fixed in PFA, and immunolabeled with anti-FLAG antibody. Scale bar: 10 µm.
(TIF)

Figure S6 **BPAG1a/b are not co-localized with FAs and do not affect FAs or C2.7 cell-substratum adhesion.** A) Control and BPAG1 knockdown C2.7 cells were cultured for 24 h on uncoated glass cover slips and processed for immunofluorescence observation. No significant differences in pattern of vinculin, a focal adhesion marker, were noted between the two cell groups. Adhesion assay was performed seeding cells into B) non-coated or C) collagen-coated 12-well dishes, respectively. Data are mean ± SEM. Student's t-test, n = 3 independent experiments.
(TIF)

Figure S7 **Expression of BPAG1a fragments containing p150^Glued^ interaction region leads to higher Golgi compactness in C2.7 myoblasts.** A) and B) Cells were transfected with the indicated constructs (see Fig. S5A), fixed in PFA, and immunolabeled with anti-GM130 antibodies. Scale bar: 20 µm. The arrows indicate transfected cells with typical Golgi compaction. White frames indicate areas magnified in A′ and B′. Note the unexpected diffuse pattern of mCherry-SR21-GAR in contrast to SR36-GAR or smaller GAR constructs (data not shown), suggesting a cis regulation of the GAR domain activity in SR21-GAR. C) Quantification of Golgi compactness was done by calculating the percentage of cells with compact Golgi out of all the total number of transfected cells found on the glass cover slip in each experiment (SR21-GAR: 5–51 cells, SR21–SR41: 9–41 cells, mCherry: 22–263 cells, non-transfected (NT): 143–1024 cells). Data are mean ± SEM. Student's *t*-test, *p = 0.019, **p = 0.004, n = 3–5 independent experiments.
(TIF)

Figure S8 **BPAG1a/b are enriched in the trailing edge of migrating C2.7 myoblasts.** C2.7 cell monolayer (80% confluent) was wounded, further incubated for 7 h and fixed. Staining with anti-BPAG1a/b serum reveals stronger signals in the back than in the front of migrating cells at the wound edge (white arrows). Black arrow indicates general direction of migration. Scale bar: 20 µm.
(TIF)

Figure S9 **Knockdown of BPAG1 reduces directness of cell migration in subconfluent C2.7 myoblasts.** A) Cells treated with siRNA, cultivated for 24 h, and reseeded at 5% confluency. Time lapse photographs were taken of control and BPAG1 KD myoblasts every 10 min for 19 h. The migratory paths of individual cells that did not divide are shown from one of the experiments. B) The mean cell velocities were measured by manual tracking using ImageJ and pooled from 2 independent experiments. Cell migration directness was quantified by calculating Euclidian distance divided by the accumulated distance for each individual cell (30 cells per siRNA condition). Data are mean ± SEM (Student's *t*-test, n = 2 independent experiments).
(TIF)

Table S1 **Primers used in this study.**
(DOCX)
