# Supplementary figures and images for: BPAG1a and b Associate with EB1 and EB3 and Modulate Vesicular Transport, Golgi Apparatus Structure, and Cell Migration in C2.7 Myoblasts
Source: PLoS One. 2014 Sep 22;9(9):e107535. doi: 10.1371/journal.pone.0107535 (PMC4171495; doi:10.1371/journal.pone.0107535)

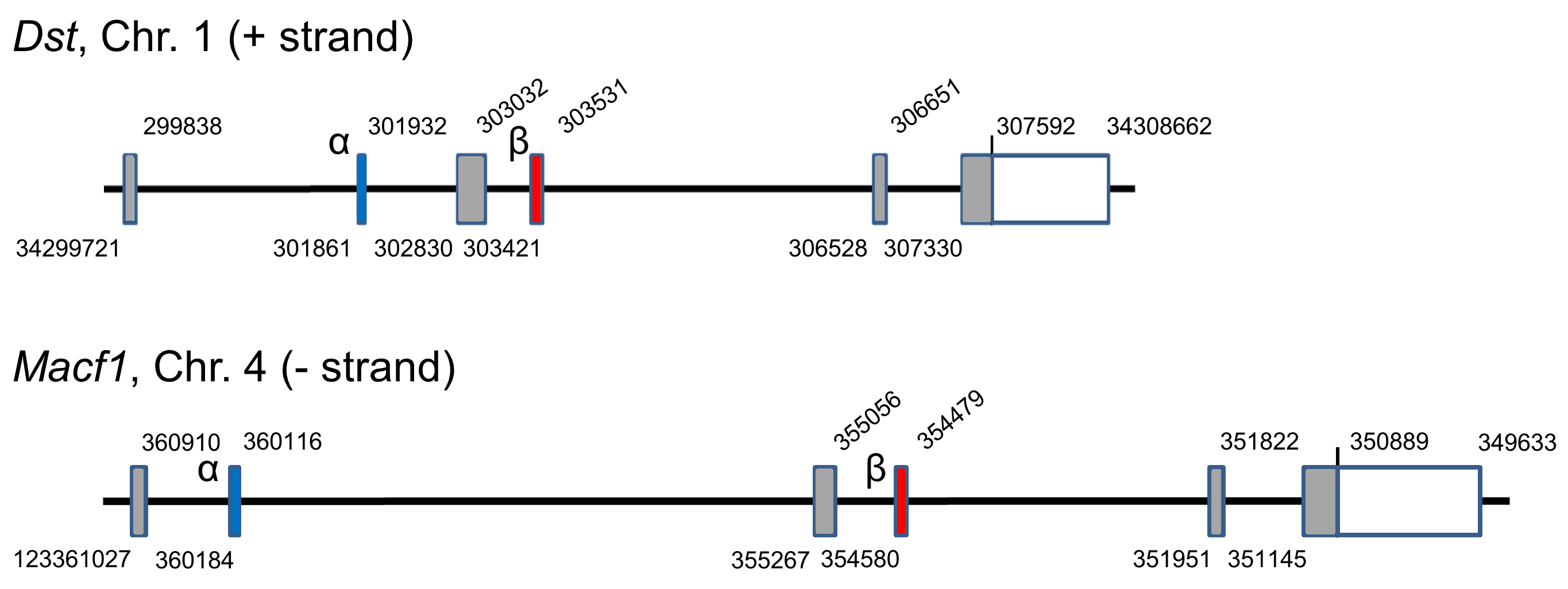

Supplement: Figure S1 — Genomic organization of the Dst and Macf1 3′ exons. The alternatively spliced exons are labeled α and β. The translated sequences are in blue, red, and grey. (TIF) [file pone.0107535.s001.tif]

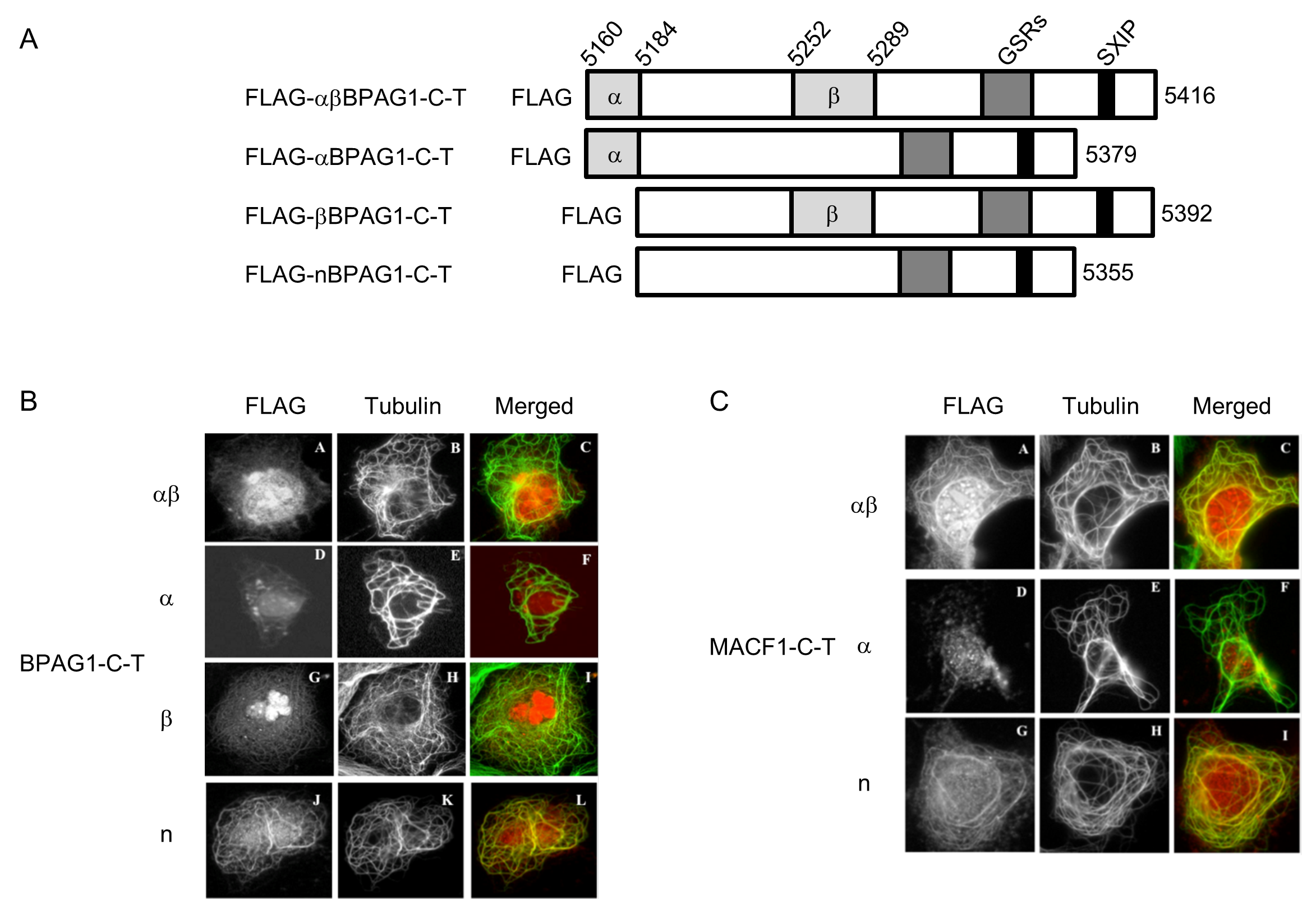

Supplement: Figure S2 — Recombinant BPAG1a/b-C-Tail isoforms and MACF1a/b-C-Tail isoforms bundle MTs in transiently transfected COS-7 cells. A) Schematic representation of FLAG-tagged BPAG1a/b-C-Tail (T) proteins expressed in transfected COS-7 cells. The amino acid numbers, corresponding to the first residue encoded by each of the last four exons, as well as the C-terminal residue of BPAG1a, are indicated (see also Fig. 2). B) Cells expressing the indicated FLAG-BPAG1a/b-C-T isoforms were double labeled with polyclonal anti-FLAG antibody (A, D, G, J) and monoclonal anti-tubulin antibody (B, E, H, K). Ectopically expressed proteins showed a predominant nuclear staining and induced bundling of MTs even though co-localization of the different BPAG1a/b-C-T isoforms with bundled MTs was not systematically observed. C) same as B) for the indicated FLAG-MACF1a/b-C-T isoforms corresponding to the respective FLAG-BPAG1a/b-C-T isoforms (see Fig. 2 for MACF1a/b exon borders). (TIF) [file pone.0107535.s002.tif]

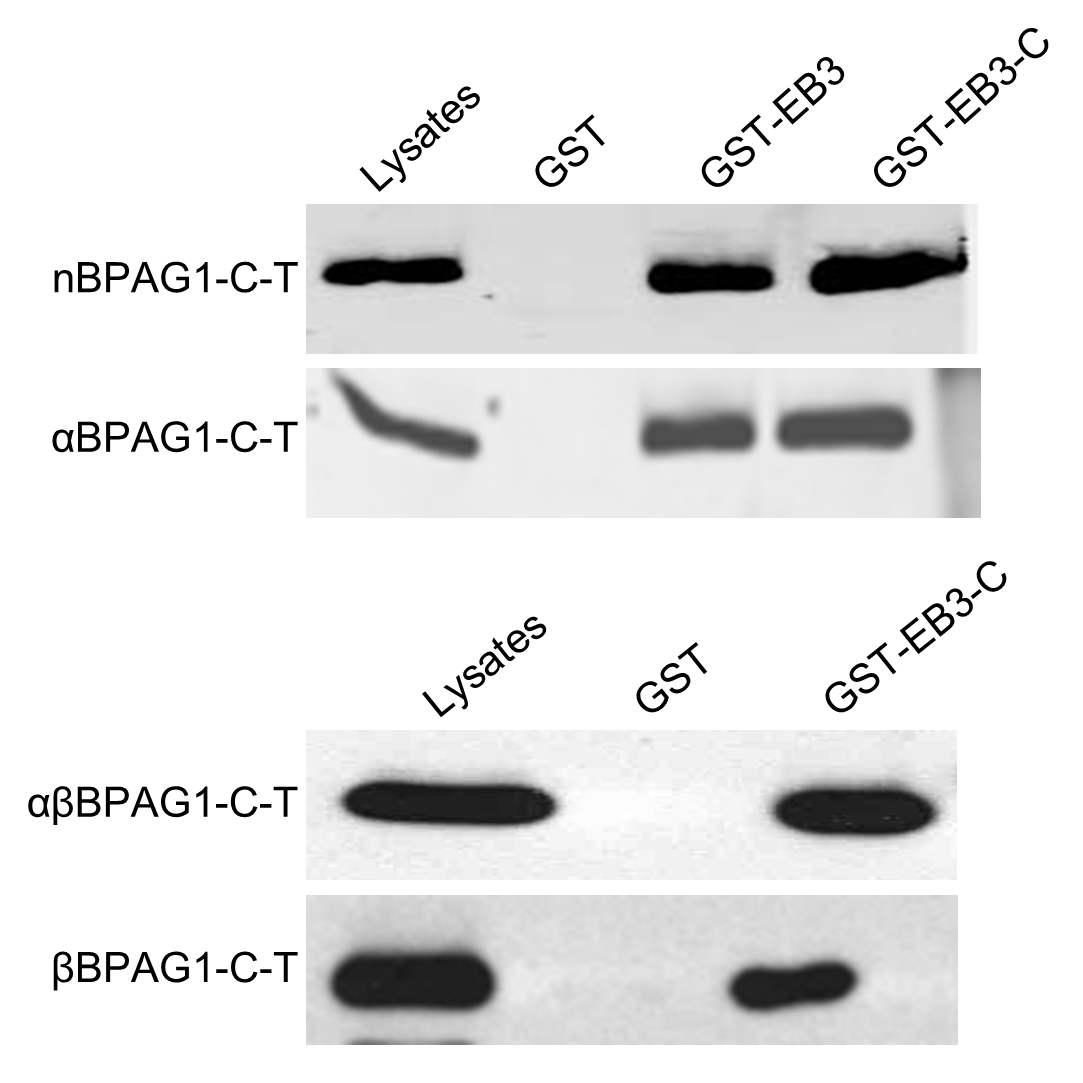

Supplement: Figure S3 — BPAG1-C-T interacts with EB3 in GST pull-down assays. COS-7 cells were transfected with different FLAG-tagged BPAG1-C-T constructs and their lysates were used in GST pull-down assays with GST, GST-EB3 (res. 1-281), or -EB3-C (res. 145-281) as indicated. The presence of FLAG-BPAG1-C-T associated with the glutathione-Sepharose beads was detected by Western blotting with an anti-FLAG antibody. GST-APC-C did not pull down FLAG-nBPAG1-C-T (data not shown). (TIF) [file pone.0107535.s003.tif]

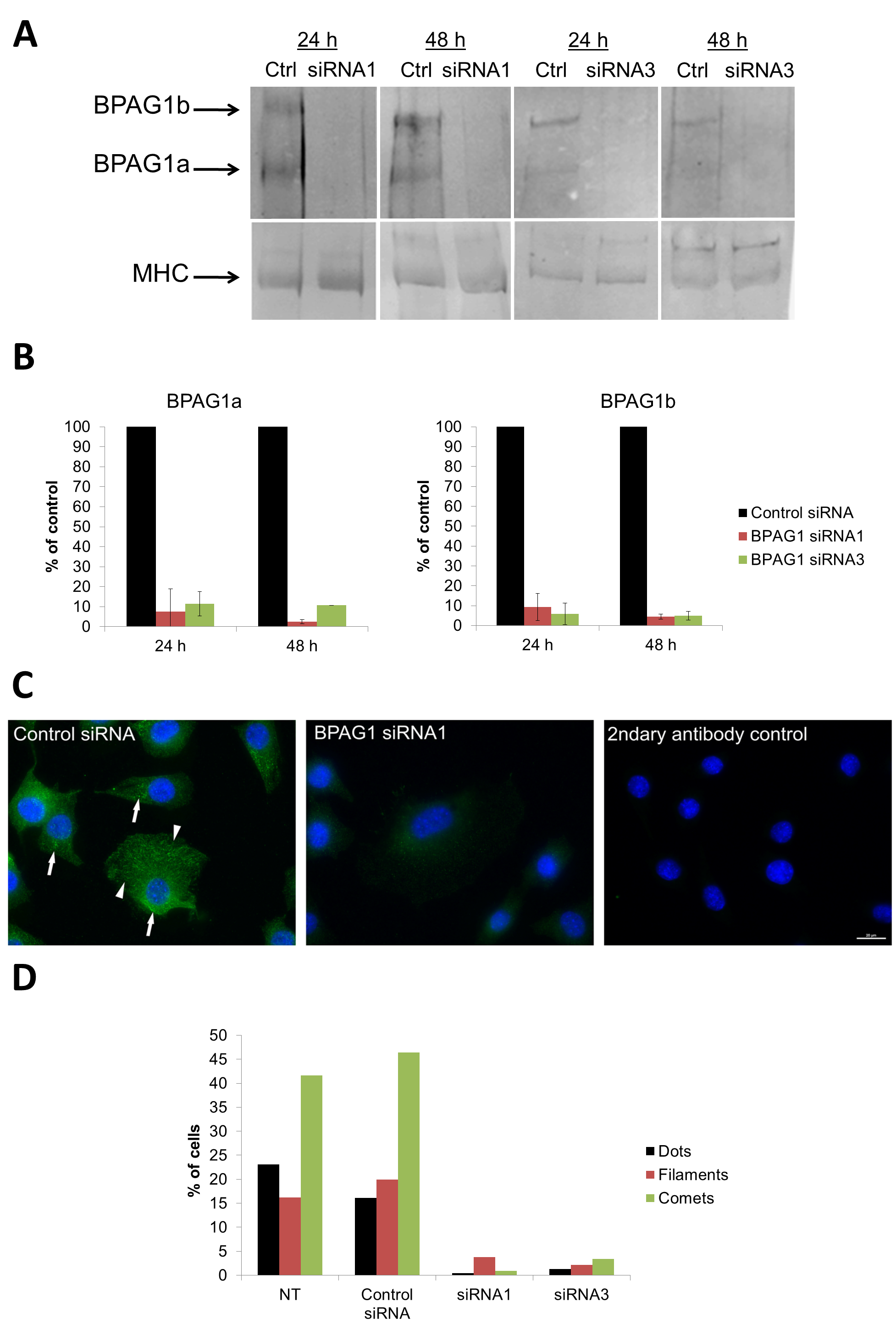

Supplement: Figure S4 — BPAG1a/b can be efficiently knocked down in C2.7 myoblasts. A) Western blot analysis of BPAG1a/b and myosin heavy chain (MHC) levels in cells transfected with control (Ctrl) siRNA or BPAG1 siRNA1 or siRNA3. BPAG1a/b was detected with anti-serum R18611 and MHC was detected sequentially on the same blot. Results are representative of three independent experiments for 24 h after transfection and two for 48 h after transfection for BPAG1 siRNA1 and siRNA3. B) Quantification of BPAG1a/b knockdown by normalization to MHC. Data are mean ± SD. C) Cells treated with control siRNA or BPAG1 siRNA1 for 24 h were fixed in PFA and immunolabeled with anti-BPAG1a/b serum R18024. Scale bar: 20 µm. BPAG1a/b have a dot-like (arrows) and short-filament-like (arrowheads) pattern. The same results were obtained for cells treated with siRNA3 (data not shown, see also Fig. 8). D) The three types of BPAG1a/b pattern were quantified in cells not treated by siRNA (NT), treated with control siRNA, BPAG1 siRNA1 or siRNA3 (n = 204–235 cells/condition). (TIF) [file pone.0107535.s004.tif]

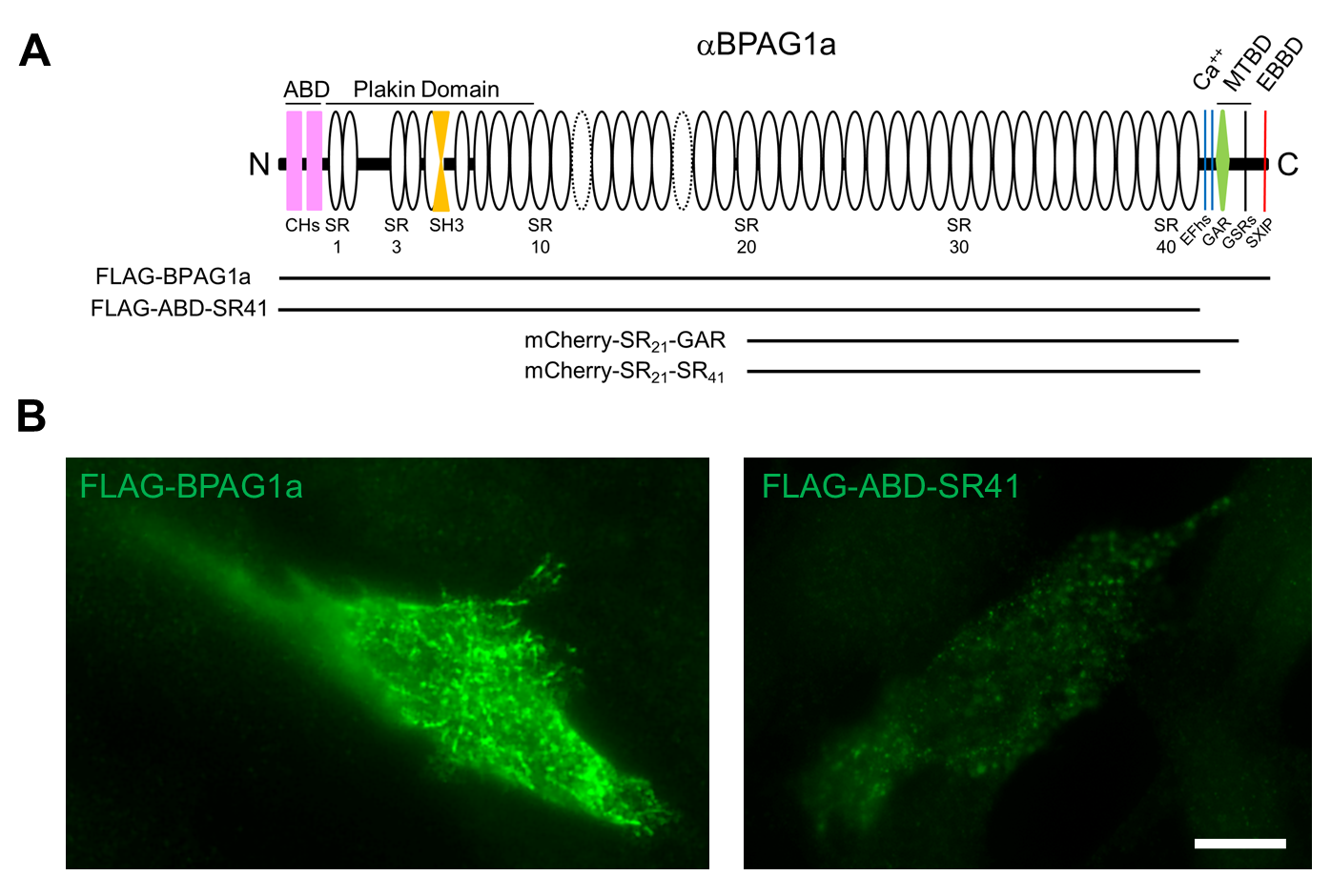

Supplement: Figure S5 — FLAG-BPAG1a and FLAG-ABD-SR41 display strikingly different patterns. A) BPAG1a constructs used to transfect C2.7 cells. The borders of the FLAG- and mCherry-tagged recombinant proteins are indicated by black lines under the scheme of αBPAG1a. B) Cells were transfected with the indicated constructs, fixed in PFA, and immunolabeled with anti-FLAG antibody. Scale bar: 10 µm. (TIF) [file pone.0107535.s005.tif]

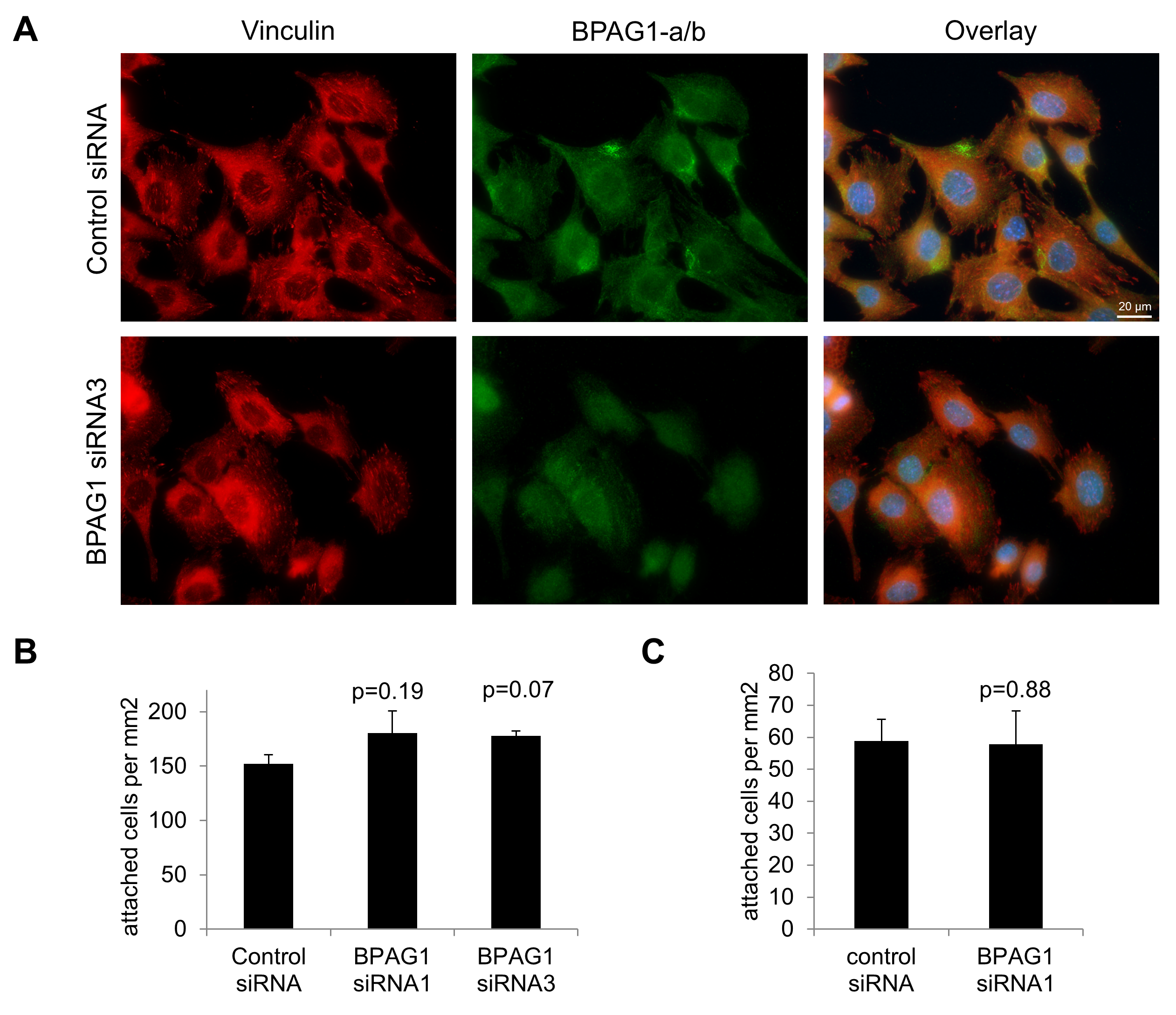

Supplement: Figure S6 — BPAG1a/b are not co-localized with FAs and do not affect FAs or C2.7 cell-substratum adhesion. A) Control and BPAG1 knockdown C2.7 cells were cultured for 24 h on uncoated glass cover slips and processed for immunofluorescence observation. No significant differences in pattern of vinculin, a focal adhesion marker, were noted between the two cell groups. Adhesion assay was performed seeding cells into B) non-coated or C) collagen-coated 12-well dishes, respectively. Data are mean ± SEM. Student's t-test, n = 3 independent experiments. (TIF) [file pone.0107535.s006.tif]

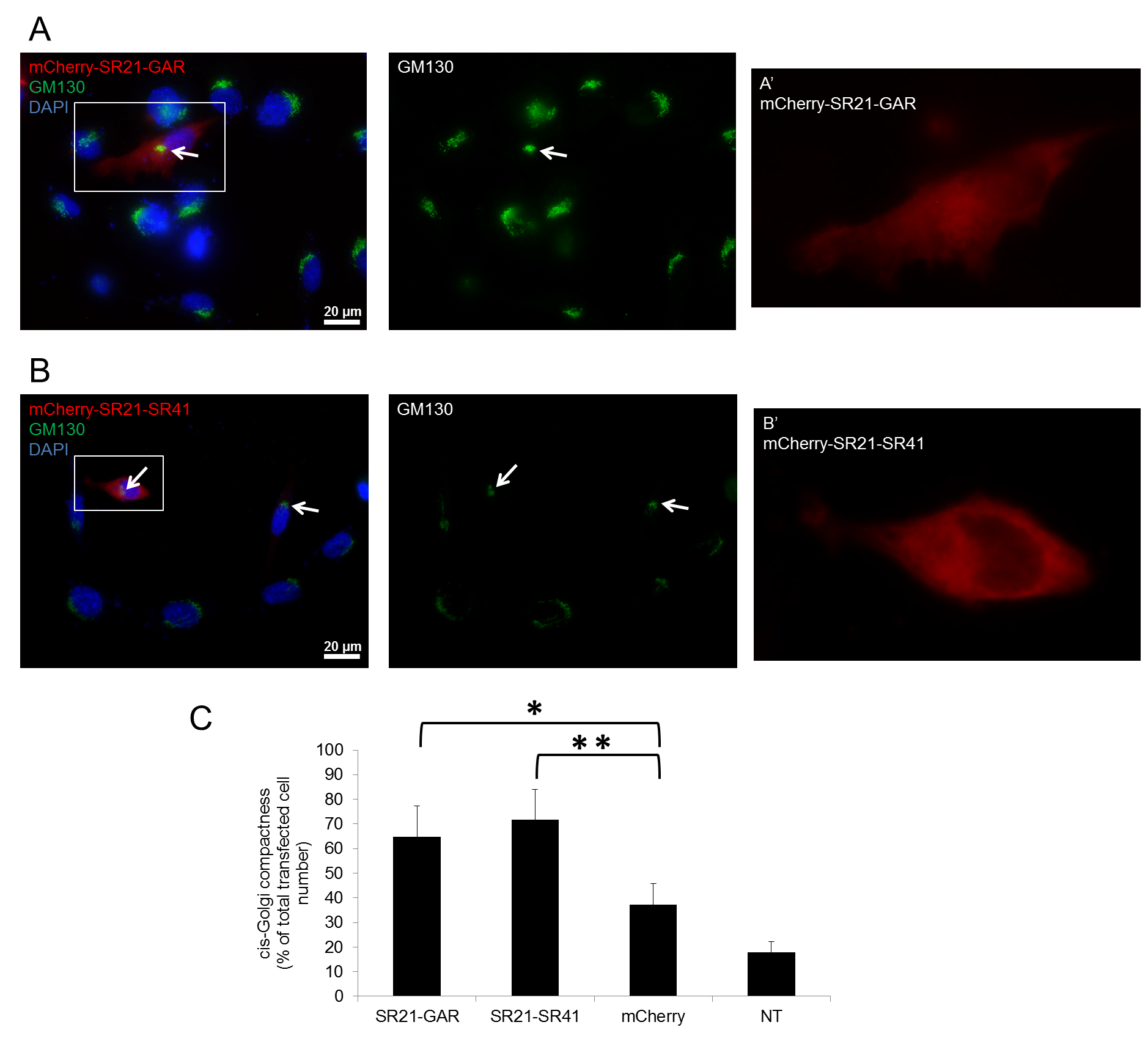

Supplement: Figure S7 — Expression of BPAG1a fragments containing p150Glued interaction region leads to higher Golgi compactness in C2.7 myoblasts. A) and B) Cells were transfected with the indicated constructs (see Fig. S5A), fixed in PFA, and immunolabeled with anti-GM130 antibodies. Scale bar: 20 µm. The arrows indicate transfected cells with typical Golgi compaction. White frames indicate areas magnified in A′ and B′. Note the unexpected diffuse pattern of mCherry-SR21-GAR in contrast to SR36-GAR or smaller GAR constructs (data not shown), suggesting a cis regulation of the GAR domain activity in SR21-GAR. C) Quantification of Golgi compactness was done by calculating the percentage of cells with compact Golgi out of all the total number of transfected cells found on the glass cover slip in each experiment (SR21-GAR: 5–51 cells, SR21–SR41: 9–41 cells, mCherry: 22–263 cells, non-transfected (NT): 143–1024 cells). Data are mean ± SEM. Student's t-test, *p = 0.019, **p = 0.004, n = 3–5 independent experiments. (TIF) [file pone.0107535.s007.tif]

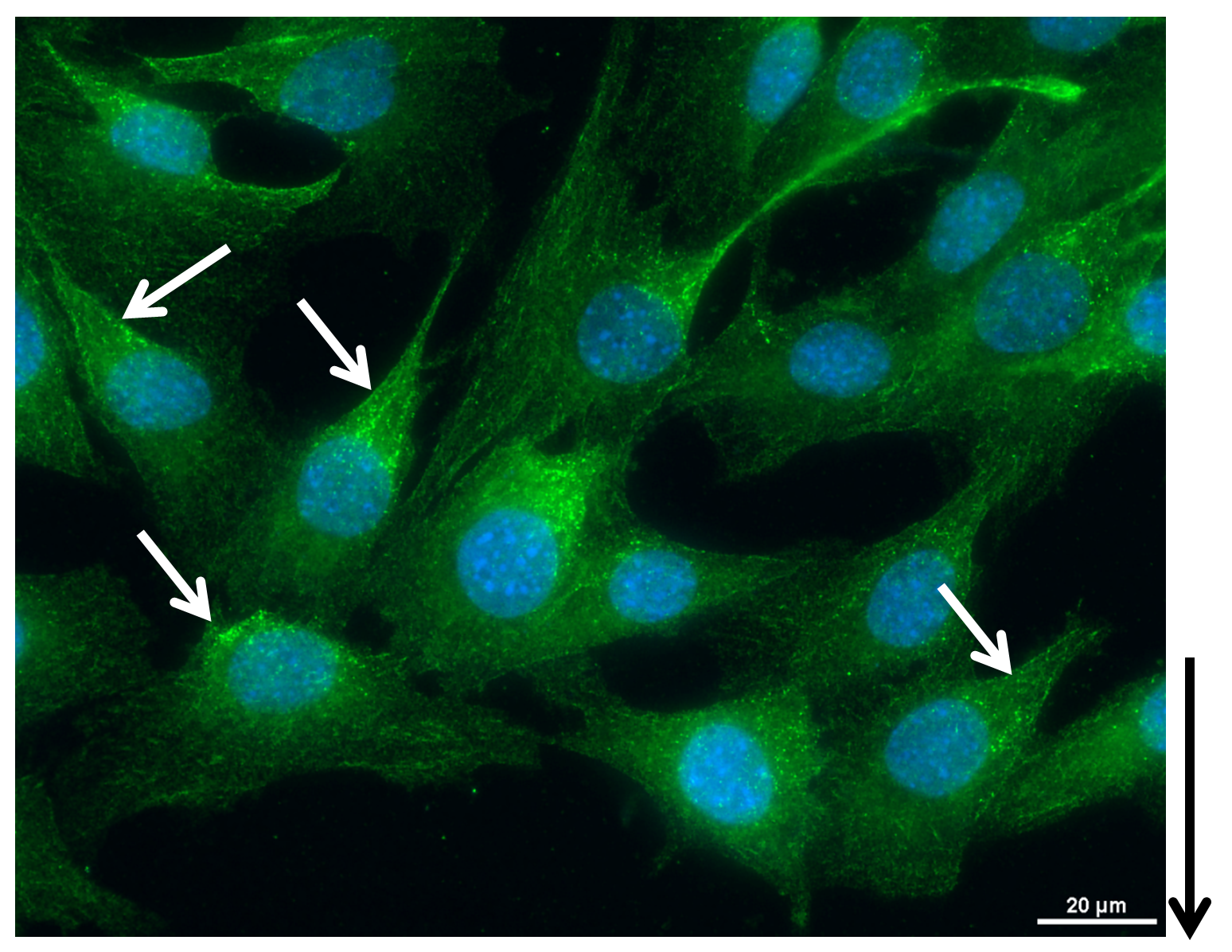

Supplement: Figure S8 — BPAG1a/b are enriched in the trailing edge of migrating C2.7 myoblasts. C2.7 cell monolayer (80% confluent) was wounded, further incubated for 7 h and fixed. Staining with anti-BPAG1a/b serum reveals stronger signals in the back than in the front of migrating cells at the wound edge (white arrows). Black arrow indicates general direction of migration. Scale bar: 20 µm. (TIF) [file pone.0107535.s008.tif]

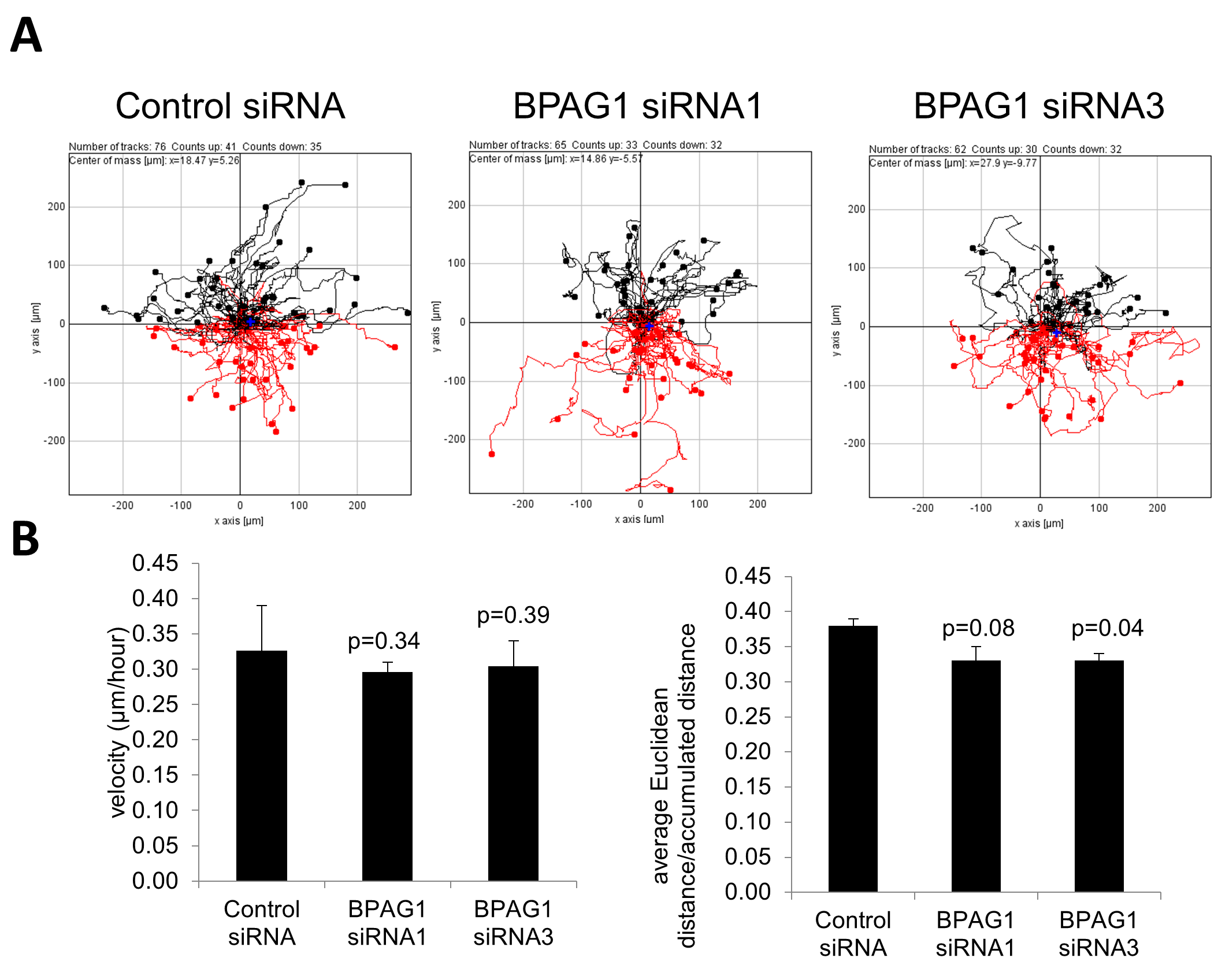

Supplement: Figure S9 — Knockdown of BPAG1 reduces directness of cell migration in subconfluent C2.7 myoblasts. A) Cells treated with siRNA, cultivated for 24 h, and reseeded at 5% confluency. Time lapse photographs were taken of control and BPAG1 KD myoblasts every 10 min for 19 h. The migratory paths of individual cells that did not divide are shown from one of the experiments. B) The mean cell velocities were measured by manual tracking using ImageJ and pooled from 2 independent experiments. Cell migration directness was quantified by calculating Euclidian distance divided by the accumulated distance for each individual cell (30 cells per siRNA condition). Data are mean ± SEM (Student's t-test, n = 2 independent experiments). (TIF) [file pone.0107535.s009.tif]
