# Supplementary material for: BPAG1a and b Associate with EB1 and EB3 and Modulate Vesicular Transport, Golgi Apparatus Structure, and Cell Migration in C2.7 Myoblasts
Source: PLoS One. 2014 Sep 22;9(9):e107535. doi: 10.1371/journal.pone.0107535 (PMC4171495; doi:10.1371/journal.pone.0107535)
Supplement: Table S1 — Primers used in this study. (DOCX) [file pone.0107535.s010.docx]

**Table S1. Primers used in this study.**

| cDNA (mouse) | Cloning primers |
| --- | --- |
| BPAG1a-C-T | 5’-GAATTCACCCTGCAGGGTTCACCATCAC-3’  5’-GATATCGGCTATCTCTTCGAAGACTTG-3’ |
| MACF1a-C-T | 5’-GAATTCACCCTGCCGAGTTCATCATCCTGGT-3’  5’-GATATCGATTATCGCTTGGGACCTGGAGT-3’ |
| EB3 | 5’-GGATCCATGGCTGTCAATGTGTACTCCACTTCT-3’  5’-GAATTCCTCAGTACTCGTCCTGGTCTTCCT-3’ |
| EB3-C | 5’-GGATCCATGATCTTCAACAAATCCAAGAAACTCATTG-3’  5’-GAATTCCTCAGTACTCGTCCTGGTCTTCCT-3’ |
| Variant | RT-PCR primers |
| αβBPAG1a | 5’-GTGAAAAATGATCCCTGCAGGGTTCACCATCACGGGAGT-3’  5’-TCCCTGGATTGGTGTGCCCTCTGCGGATGACACGGGAAA-3’ |
| αBPAG1a | 5’-GTGAAAAATGATCCCTGCAGGGTTCACCATCACGGGAGT-3’  5’-CTTGCTTCCCTGGATTGGTGTGCCCTTGGGTGTGCTGGC-3’ |
| βBPAG1a | 5’GTGAAAAATGATCCCTGCAGGGCTAAAGGAAGGACCAAC-3’  5’-TCCCTGGATTGGTGTGCCCTCTGCGGATGACACGGGAAA-3’ |
| nBPAG1a | 5’-GTGAAAAATGATCCCTGCAGGGCTAAAGGAAGGACCAAC-3’  5’-CTTGCTTCCCTGGATTGGTGTGCCCTTGGGTGTGCTGGC-3’ |
| αβMACF1a | 5’-GTGAAAAATGACCCCTGCCGAGTTCATCATCCTGGGAGT-3’  5’-GCCTGATGACGAAATAACCTCAGGGCTGAGGAGCTGGAG-3’ |
| αMACF1a | 5’-GTGAAAAATGACCCCTGCCGAGTTCATCATCCTGGGAGT-3’  5’-GCTGCCTGATGACGAAATAACCTTGGTCCCGCTGGCTGG-3’ |
| βMACF1a | 5’-GTGAAAAATGACCCCTGCCGAGCACGAGGCAGAACTAAC-3’  5’-GCCTGATGACGAAATAACCTCAGGGCTGAGGAGCTGGAG-3’ |
| nMACF1a | 5’-GTGAAAAATGACCCCTGCCGAGCACGAGGCAGAACTAAC-3’  5’-GCTGCCTGATGACGAAATAACCTTGGTCCCGCTGGCTGG-3’ |
